# Making Use of Si Contaminants during Chemical Vapor Deposition of Graphene on Cu: Synthesis of a Stable Material with the Textbook-like Band Structure of Free-Standing Graphene

**DOI:** 10.1021/acsami.5c06939

**Published:** 2025-07-02

**Authors:** Tim Kratky, Jürgen Kraus, Paul M. Leidinger, Patrick Zeller, Francesca Genuzio, Alessandro Sala, Tevfik Onur Menteş, Andrea Locatelli, Sebastian Günther

**Affiliations:** † Department of Chemistry, Physical Chemistry with Focus on Catalysis, 9184 Technical University of Munich (TUM), Lichtenbergstr 4, Garching 85748, Germany; ‡ Catalysis Research Center, Ernst-Otto-Fischer-Street. 1, Garching 85748, Germany; § Elettra-Sincrotrone Trieste S.C.p.A., S.S. 14—km 163,5 in Area Science Park, 34149 Basovizza, Trieste, Italy

**Keywords:** graphene, chemical vapor deposition, copper, silica, angle-resolved photoelectron spectroscopy, band structure, spectroscopic photoelectron and low energy electron microscopy

## Abstract

We report on a gentle procedure for the complete electronic decoupling of graphene from Cu. The procedure can be added to the growth protocol of graphene synthesis by chemical vapor deposition making use of the widely unnoticed silicon release from hot wall quartz tube reactors. So far, Si release was observed if the effect was large, so that it deteriorates the grown graphene. However, the effect can also be used to turn the electronic band structure of CVD-grown graphene on Cu into that of free-standing graphene as shown in a combined spectroscopic photoelectron and low energy electron microscopy study. Adding a foil pretreatment to the synthesis protocol in the reactor turns the polycrystalline foil into (111)-textured Cu, and the electronic band structure of CVD-grown graphene on Cu(111) is achieved with n-doping by −0.4 eV and band gap formation of 0.3 eV. If, however, graphene is synthesized on a Si-loaded Cu foil, subsequent oxygen exposure in the reactor segregates the dissolved Si to the surface and converts it to intercalated silica without destroying the covering graphene. The graphene decouples electronically, and the textbook-like electronic band structure of free-standing graphene emerges. The alternating stacking of 30°-rotated layers in thicker graphene leads to electronically noninteracting layers. Moreover, in angle-resolved photoemission, replica bands due to Umklapp processes emerge without the opening of an energy gap.

## Graphical abstract

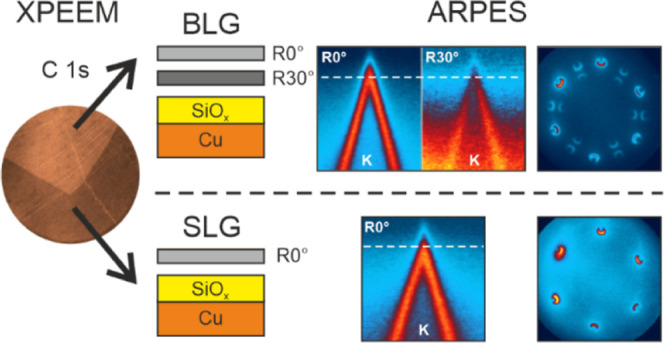

## Introduction

One of the promising synthesis routes of graphene (g) capable of mass-scale production is the catalytic chemical vapor deposition (CVD) on transition metal (TM) supports.^1–3^ However, the presence of a support influences the properties of the synthesized material. Depending on whether there are chemical bonds between the carbon layer and the underlying substrate or whether the material is physisorbed, the electronic properties greatly change, and thus, g/TM heterostructures are divided into strongly and weakly interacting systems.^4–9^ In the case of a strong interaction, the degree of hybridization of the support and graphene can be chemically altered by intercalation of a foreign material. In addition to the support itself, the covering graphene layer may also be chemically modified to change the properties of the supported graphene. Both techniques are applied during the so-called “chemical bandgap engineering”.^7^ However, also in weakly interacting g-TM systems, the properties of the graphene layer are altered by the underlying support. The mismatch of graphene and support lattice induces mechanical strain and leads to moiré pattern formation.^8^ Since charge redistribution also appears during physisorption, all mentioned effects influence the electronic band structure and are used in “physical band gap engineering”.^7^ Of course, also the mechanical properties are influenced by the presence of the support (even in weakly interacting systems) as intercalation may lead to an altered adhesion and chemistry below the synthesized graphene layer.^10^ For example, graphene acts as a very effective oxidation protection layer for Cu on the day-to-week time scale, but this protection surprisingly worsens in the long run.^11^

The effects described above are readily observed when investigating the valence band structure with the help of angle-resolved photoelectron emission spectroscopy (ARPES). Graphene on strongly interacting TM supports, such as g/Ni(111),^12,13^ g/Co(0001),^14^ g/Fe(110),^15^ or g/Ru(0001),^16,17^ displays an altered π-band in the vicinity of the *K*-point: Instead of an intact Dirac point, a large energy gap evolves below the Fermi energy due to hybridization of graphene orbitals with the TM support. The degree of hybridization is greatly reduced when intercalating oxygen^17,18^ or an inert metal like gold^13,15^ between graphene and the support so that the Dirac cone is restored at the *K*-point. In weakly interacting g/TM systems, such as g/Ir(111),^19^ g/Pt(111),^20^ or g/Cu(111),^21^ the Dirac cone of free-standing graphene is only slightly altered due to the presence of the support. However, p-doping^19,20^ or n-doping^21^ of the electronic bands may be induced by charge transfer to or from the support, an energy gap at the *K*-point may^21^ or may not be observed,^19,20^ or replicas and mini-gaps^19^ may occur due to the presence of moiré beating of the graphene and support lattice. Thus, it is no surprise that intercalation affects these latter parameters, irrespective of the interaction strength between graphene and the support material.

Various strategies for how to protect the synthesized carbon layer have been under investigation. A radical way is to detach the synthesized graphene layer from the catalytic support and transfer it onto a suitable, more inert support^22–24^ or even to encase it by a covering layer such as h-BN.^25,26^ Another approach is to convert the catalytic support into an inert interface by intercalation and chemical conversion in order to achieve long-term stability of the supported graphene.^18,27^ One route used a metallic support that develops an oxide layer by oxygen intercalation without attacking the covering graphene layer.^28^ However, this approach used highly reactive metal alloy samples that require ultrahigh-vacuum (UHV) conditions, i.e., the support cannot be transferred through air without deterioration.

In an alternative preparation route, a foreign material, such as Si, is evaporated on a g/TM sample where it intercalates and converts into an unreactive oxide in the presence of adsorbing oxygen.^29,30^ However, the successful proof of concept was conducted in UHV. The evaporation of Si is not easy to implement in a CVD reactor, and the intercalation of Si on metal supports underneath graphene typically requires point defects or even terminating edges of the graphene layer,^31^ which provide access to the support so that sufficient foreign material can intercalate and homogeneously distribute under the covering graphene layer before conversion into an oxide upon oxygen adsorption.

In this publication, we report a straightforward procedure on how to avoid such difficulties when decoupling graphene from supporting Cu. The outlined protocol makes use of a well-known silicon contamination of the Cu foil during graphene CVD growth in quartz tube reactors which results from the release of SiO from the reactor wall at high temperatures and reductive atmospheres.^32–36^ Recently, it was observed that the contaminating Si does not directly develop SiO_*x*_ particles that deteriorate the grown graphene film, which must be avoided. Instead, the released SiO species can be reduced on the support so that Si dissolves in the Cu bulk reaching a concentration close to or below the X-ray photoelectron detection limit.^37^ The Si contamination often occurs unintentionally when Cu foils are pretreated at high temperature and elevated H_2_ pressure to turn the polycrystalline Cu support into a (111)-textured foil, conditions that also release SiO from the reactor wall.^38–41^ Depending on the following treatment, Si can be gently segregated to the g/Cu interface when adsorbing oxygen. The conversion of the segregated Si toward a thin surface silica layer consumes the adsorbing oxygen, making the graphene layer seemingly oxygen-resistant.^37^ The existence of a silica layer in the presence or absence of oxygen might also explain why certain preparation recipes of graphene are successful as they affect the catalytic activity of the Cu support.^42–44^ We used this effect to gently decouple CVD-grown graphene on Cu from the support during synthesis in a quartz tube reactor. In a pretreatment step, the Cu foil is Si-enriched, followed by the CVD growth of graphene on metallic Cu. When the grown graphene is subsequently exposed to a low oxygen partial pressure, a thin silica film is grown at the g/Cu interface, which electronically decouples the carbon layer from the metal support. The result is graphene with the textbook band structure of free-standing single-layer graphene (SLG) as observed by ARPES. We can show that this property is also conserved for bilayer (BLG) and trilayer graphene (TLG) if each subsequent layer is rotated by 30° with respect to each other. The released photoelectrons, however, scatter on their way to the detection system so that they may pick up reciprocal lattice vectors, resulting in ARPES signatures of 30°-rotated SLG sheets. The data are compared to those obtained from CVD-grown graphene on (111)-textured metallic Cu, where layer-dependent doping and the formation of a band gap are observed.

## Results and Discussion

### Properties of CVD-Grown Graphene on Metallic (111)-Textured Cu Foils

The CVD synthesis of graphene on Cu in a hot wall quartz tube reactor leads to Si release from the reactor wall at hydrogen pressures of >100 mbar and elevated temperature. The released Si dissolves in the Cu foil and may segregate to the surface in the presence of oxygen at temperatures above 250 °C. When aiming to synthesize graphene interacting with metallic Cu, the dissolution and segregation of Si must be avoided. To achieve this, the Si release from quartz components during CVD synthesis may be minimized, and more importantly, Si segregation must be suppressed. Potential intercalants originating from contact to air are removed by degassing under vacuum. However, degassing must be performed at temperatures below 250 °C. This low-temperature annealing is essential before applying any higher temperature where dissolved Si gets mobile because it guarantees the desorption of potential intercalants such as oxygen and water that might trigger Si segregation by silicon oxide formation at the Cu foil surface.^37^ Following this line, graphene was synthesized on a metallic (111)-textured Cu foil trying to minimize the amount of segregated Si as outlined in the Supporting Information. The synthesized graphene was a monocrystalline SLG film entirely covering the Cu foil. Locally, the film contained bilayer and trilayer islands (BLG and TLG). The sample was thoroughly degassed under vacuum until only a negligible amount of oxygen and silicon was observed and investigated by low-energy electron microscopy (LEEM) as summarized in the Supporting Information (see Figure S1). The locally characterized SLG region on a Cu(111) grain showed a graphene lattice rotated by 2.5° with respect to the Cu lattice, leading to typical moiré patterns in reciprocal space data.^45^ The characterized BLG region consisted of two stacked graphene lattices rotated by 30° with respect to each other. The SLG and BLG nature of the graphene film was identified by the characteristic quantum oscillation of the electron reflectivity at low kinetic energy.

Figure 1 displays angle-resolved photoemission spectroscopy (ARPES) data taken from the SLG and BLG regions of the sample. The experiments were conducted with 50 eV photon illumination at an energy resolution of 0.30 eV (see the Experimental Section). In order to visualize low- and high-intensity features within the same plot, a false color table is used with dark to light blue visualizing increments at low intensity and red to yellow color the intensity increase at a high level. Figure 1a,b displays momentum plots of SLG and BLG taken at the indicated photoelectron binding energy. The plots show data from which the linear chromatic aberration has been subtracted using a tool described in the literature^46^ without application of further symmetricity procedures.

**Figure 1:**
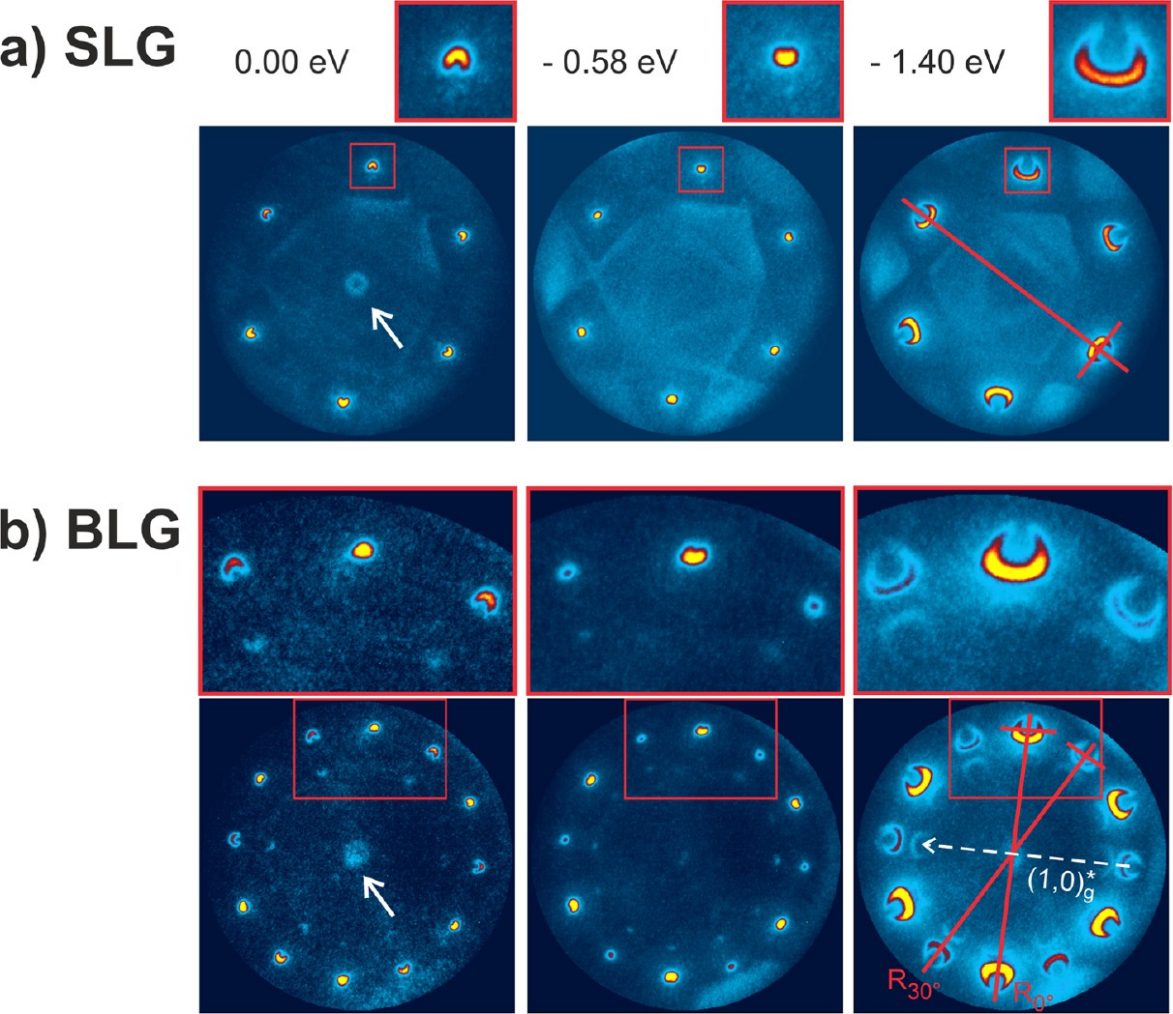
Momentum plots of SLG and BLG taken on the Cu(111) grain of the supporting foil acquired at the indicated electron binding energy. (a) Photoemission data of SLG shows the dispersion of the Dirac cone at the *K* point (see insets). The SLG data reveals photoelectron emission intensity originating from the Cu(111) support, e.g., the white arrow points to electron emission from the Cu(111) surface state which also appears under BLG (see text). (b) Equivalent photoelectron emission data taken from BLG. Replica spots by photoemission from the R30° graphene lattice are observed. The dashed (1,0)*g vector of the unrotated graphene lattice indicates the translation causing the intense replicas in the R30° data of the momentum plots (see the text and the construction in the reciprocal space shown in Figure 7). The red lines in the lower right panel indicate the directions of the 0°- and 30°-rotated carbon lattices along which the photoelectron intensities were extracted (see Figure 2) (Videos S1 and S2 of the data set are given in the Supporting Information). Photon energy = 50 eV.

The red boxes in the upper part of the respective momentum plots of Figure 1a point out the photoelectron emission intensity at one of the six *K* points of the reciprocal SLG lattice. The top insets display magnifications of this region. The U-shaped photoelectron emission feature surrounding the *K*-point represents a constant binding energy cut through the graphene Dirac cone.^47^ The dispersion of the band feature as a function of binding energy can be judged from the three images from 0.00 to −1.40 eV, which indicates that the Dirac point of SLG is located somewhat below Fermi energy. In other words, the analyzed SLG layer is n-doped. (Video S1 of the data set can be found in the Supporting Information.)

All other diffuse features of the momentum plots arise from the band structure of the Cu(111) support with its characteristic 3-fold symmetry which reflects the Fermi surface of an fcc crystal (see the left panel of Figure 1a).^48^ A white arrow points to a circle at the Γ-point in the center of the momentum plot. This feature is caused by photoelectron emission of the Cu(111) surface state. As already observed by Walter et al., the Cu(111) surface state persists, although the Cu foil is covered by SLG.^21,49^ The surface state is connected to the electron accumulation at about 1 Å in front of the Cu(111) surface, whereas the SLG layer is situated far outside, explaining the low degree of electronic coupling.

Umklapp processes and the occurrence of mini gaps reported for g/Ir(111)^19^ are not observed here. This finding again points toward the absence of a periodic structural corrugation in the graphene layer. In other words, the moiré spots seen in the LEED pattern of Figure S1 of the Supporting Information are likely caused by double scattering between the mismatching Cu and graphene lattices. Summing up, the photoelectron emission data of SLG/Cu(111) in Figure 1a show the rather low degree of electronic interaction between the SLG and the Cu(111) foil surface. Due to the absence of moiré-induced features, we neglect the slight rotation of the graphene layer by 2.5° with respect to the Cu(111) support and abbreviate the orientation of the SLG as R0° in order to simplify the following discussion of ARPES data taken from the BLG region on Cu(111).

Figure 1b displays momentum plots acquired from BLG equivalent to the data taken from the SLG region on Cu(111) (Figure 1a). The Cu(111) surface state is still present but can be barely observed due to the increased signal attenuation caused by the covering BLG (see white arrow), while other features originating from the Cu(111) support are not visible anymore. Instead, prominent photoelectron emission from the covering BLG film is recorded. The 6 prominent band structure features originating from the topmost graphene layer are observed at the same *k*-space positions as in the momentum plots of SLG shown in Figure 1a. In addition, similar emission features arise from the 30°-rotated second carbon layer in BLG appearing at the 30°-rotated *k*-space positions, accordingly. Insets display the zoomed regions similar to the ones shown in Figure 1a. According to the stacking sequence of BLG with its known inverted wedding cake geometry,^50^ pronounced photoelectron emission is observed from the top, nonrotated layer (R0°—center spot of the insets). In contrast, the photoelectron emission from the lower, 30°-rotated lattice is strongly attenuated, resulting in the weak intensities at the rotated *K*-point position (R30°—left and right spots of the insets). An inspection of the constant binding energy cuts in the insets suggest that the electronic band structures of the R0° and R30° layer in BLG differ from one another regarding the energy position of the Dirac point. As seen with the help of Figure 2, the property stems from the different doping levels of the two stacked carbon layers in BLG.

**Figure 2:**
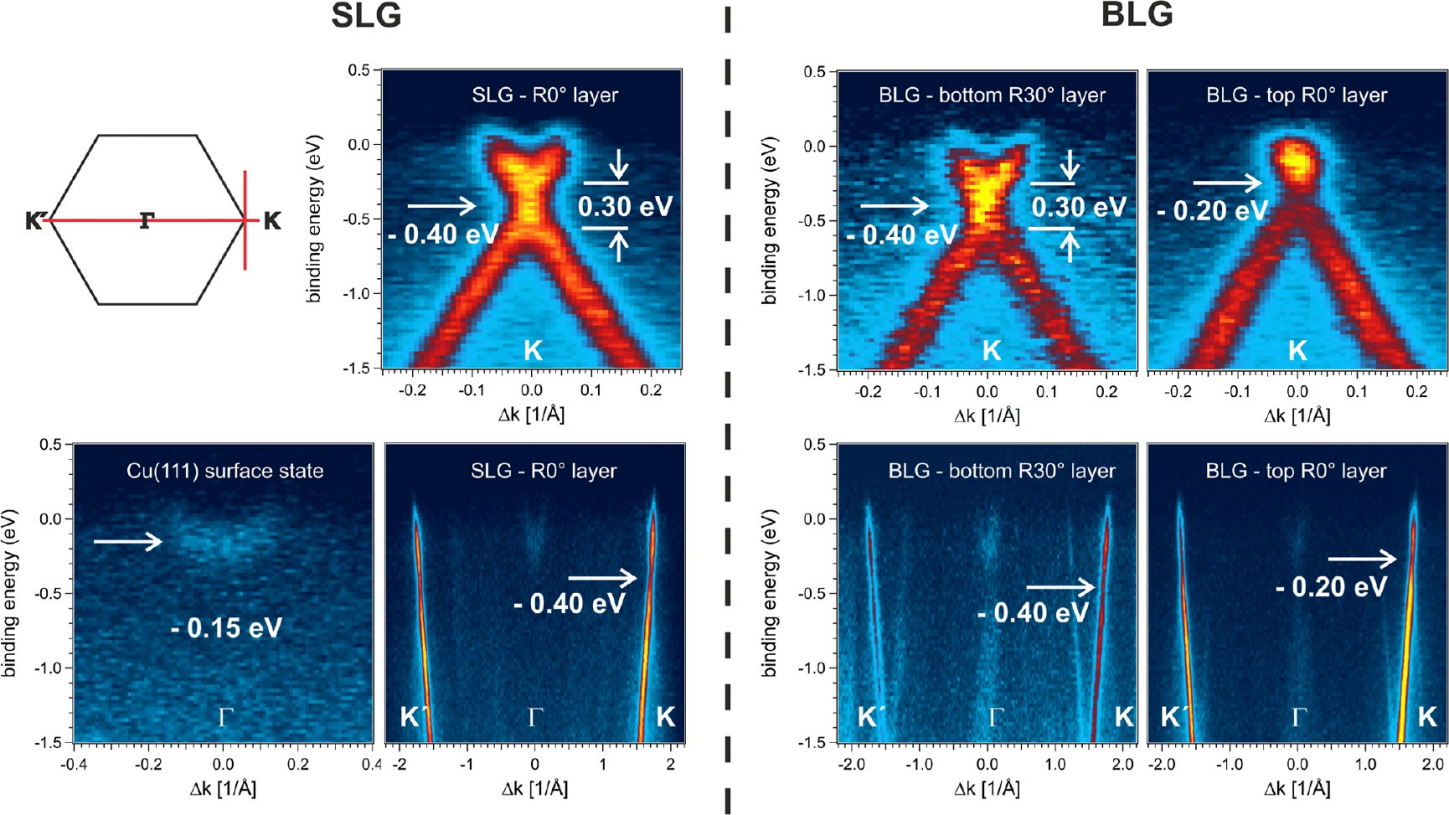
Left panel: Sketch of the first Brillouin zone of graphene. Directions are indicated from which the ARPES data of SLG and BLG were extracted (see Figure 1). The data relate to the band structure along ***K*** ^′^-Γ-**K** (lower row data) and along the perpendicular direction at the ***K***-point (upper row). Upper row data: The n-doping level at −0.40 eV and the energy gap of ∼0.30 eV are observed for SLG and the R30° layer of BLG, whereas the top R0° layer in BLG is less doped (see text). Lower row data along ***K*** ^′^-Γ-**K**: Zoom of the region at Γ reveals photoelectron emission from the Cu(111) surface state with its parabolic band dispersion. The strict linear dispersion of the π-band along ***K*** ^′^-Γ-***K*** is observed for SLG and both layers in BLG below the Dirac point with a Fermi velocity of (1.1 ± 0.1)·10^6^ m/s. Arrows indicate the position of the electronic gap in the respective panels. Note that photoelectron emission from the Cu(111) surface state at Γ is also observed under BLG. Intense replica bands appear along the ***K*** ^′^-Γ-**K** direction of the bottom R30° layer in BLG, whereas they are very faint for the top R0° graphene layer (see text).

Furthermore, replica spots are observed in the momentum plots along the main directions of the 30°-rotated lattice in BLG, which originate from the Umklapp processes. In the lower right panel of Figure 1b, a white vector sketches the required momentum transfer of one of the (1,0)*g reciprocal vectors of the unrotated graphene lattice which leads to the replica formation in the first Brillouin zone. Note that photoelectrons ejected from the lower R30° layer in BLG diffract from the topmost graphene layer on their way out, at which point they can pick up the reciprocal lattice vector of the R0° lattice. Also, note that replica formation is greatly reduced in intensity for the band structure features of the R0° lattice. Here, the equivalent Umklapp process could appear only if the photoelectrons emitted from the top graphene layer travel into the surface, diffract from the lower-lying R30° graphene layer in the backward direction, and then cross the top graphene layer on the way out. Lower backscattering cross section compared to that of forward scattering as well as the signal damping caused by the penetration through the top graphene layer explains why such replica signals are extremely weak.

The electronic band structure of SLG and BLG on Cu(111) is investigated in detail when extracting the photoelectron emission intensity as a function of the photoelectron binding energy along the *K* ^′^-Γ-*K* direction and perpendicular to it at the *K*-point as indicated by the red lines in the right panels of Figure 1a,b. Instead, due to the two 30°-rotated carbon layers in the probed BLG region, the corresponding data for the rotated layer are extracted by an additional pair of profiles, as highlighted by the red lines in the right momentum plot of Figure 1b. The derived data are compiled in Figure 2 for SLG and BLG (lower row along the *K* ^′^-Γ-*K* direction and upper row perpendicular to it at the *K*-point). The left panel of Figure 2 sketches the first Brillouin zone and the directions in the *k*-space, which relate to the displayed band structure data. The top row panels of Figure 2 prove the strictly linear dispersion of the π-band below the Dirac point in the SLG and in both layers of the BLG. Close inspection of the SLG data verifies the existence of a band gap. The modest energy resolution of the experiment smears out the gap, but the band dispersion above and below the Dirac point clearly leads to two distinct crossing points, which are vertically displaced so that a gap energy of 0.30 eV can be estimated as indicated in the graph (see Figure S2 of the Supporting Information). The midgap position at −0.40 eV locates the Dirac point at the *K*-point where the crossing of two linear bands would occur in gapless freestanding graphene. In summary, we can conclude that SLG on clean Cu(111) is n-doped with the downward energy shift of the observed −0.40 eV and develops an energy gap of about 0.30 eV at the *K*-point which fits well to the reported n-doping of graphene on copper between −0.3 eV and −0.5 eV and an energy gap of about 0.25 eV.^21,51^

Comparison with the energy bands of BLG shows an almost identical doping and gap formation in the band structure of the bottom R30° layer of BLG. Instead, the top R0° layer in BLG is less n-doped, with a doping level of −0.20 eV, in agreement with the reported decrease of n-doping with increasing graphene layer thickness.^51^ The energy gap at the *K*-point of the top R0° lattice seems to amount to about the value found for the R30° lattice. However, the precise value cannot be determined due to the upward shift of the energy band and its vicinity to the Fermi level; it cannot be decided whether the different doping levels and the intensity of the observed Umklapp process affect the size of the energy gap.

The lower row of Figure 2 displays the photoelectron emission intensity plots along the *K* ^′^-Γ-*K* direction as a function of binding energy, which reflects the band structure of the two-dimensional electronic system. The π-band proceeds strictly linearly below the Dirac point for SLG and both lattices of the BLG region, which relates to a Fermi velocity of (1.1 ± 0.1)·10^6^ m/s. When approaching the *Ḱ*- and *K*-point, the presence of the energy gap at −0.40 eV is well resolved (see white arrow) by an intensity drop and the fact that the energy bands above and below the Dirac point are offset in energy (see Figure S2). The observed band structure of R30°-stacked BLG on Cu(111) differs from the similarly R30°-stacked BLG on SiC(0001).^52^ On SiC(0001), both graphene layers are reported to be equally n-doped, and both layers show the same energy gap. Moreover, the band dispersion in the case of SiC proceeds at a slightly reduced Fermi velocity of about 0.9 × 10^6^ m/s compared to the value we report here. One may speculate that the observed differences are a consequence of the buffer layer below the BLG on SiC(0001). Another important distinction between the R30°-stacked graphene layer in BLG on Cu(111) and on SiC(0001) relates to the precision of the rotational alignment. On SiC(0001), many more replicas of the Dirac cone are observed than the ones observed in Figure 1. The authors claim that the two layers in BLG/SiC(0001) are precisely rotated by 30° with deviations below a thousandth of a degree as larger deviations would suppress the replica formation. Here, the precision regarding the 30° rotational alignment of the two layers in BLG on Cu(111) is much inferior and, consistent with the conclusions in,^52^ we do observe less replicas.

Another difference in the ARPES data emerges from the Cu foil substrate. Two of the intensity cuts shown in Figure 1 pass through the Γ-point and deliver the parabolic energy dispersion of the surface state of Cu(111). This is seen in the magnified *k*-space area of the SLG data at Γ in the left panel of the lower row in Figure 2. The equivalent photoelectron emission originating from the Cu(111) surface state is also observed underneath the BLG within the precision of the experiment. Note that the band minimum of the surface state at Γ is located at −0.15 eV instead of its known position at about −0.4 eV on clean Cu(111).^53^ Thus, we conclude that the n-doping of the carbon layer in contact with the Cu foil is caused by electron transfer from the Cu(111) surface state to graphene, explaining the up- and downward shift of the respective energy bands, which was pointed out already by Walter et al. and Gottardi et al.^21,49^ The fact that the outermost graphene layer in BLG is considerably less n-doped agrees with the findings reported by Peng et al.^51^ Note that the electron transfer from the Cu(111) substrate to the covering graphene layer does not necessarily induce the formation of a simple surface dipole, which would increase the work function. Since the opposite is reported with a work function decrease from 4.9 eV for clean Cu(111) to about 4.7 eV when being covered by graphene,^54^ the vertical charge distribution of the system most likely resembles the one observed for graphene on Ru(0001) where the net charge transfer from the substrate toward the covering graphene is not simply accumulated above but distributed above and below the graphene layer, which leads to a nondipole-like charge distribution.^55^

It cannot be judged whether the observed energy gap formation in SLG is connected to the electron transfer from the Cu(111) surface state because subtle effects of lateral interactions may or may not play a role. It was already noted that very short moiré *k*-space frequencies arise between the R0° graphene and the R-2.5 °Cu(111) lattice due to their similar lattice constants (see the LEED pattern in Figure S1 of the Supporting Information) which may not be resolved in the ARPES data but induce the formation of an energy gap. However, the same energy gap of ∼0.30 eV is also observed for the R30° graphene layer of BLG in contact with the Cu support where other moiré frequencies will occur. Thus, subtle effects caused by the slight mismatch of the support and graphene lattice must occur on a local scale, breaking the band structure symmetry at the *K* ^′^- and *K*-points, which induces the formation of an electronic band gap. Since the energy gap of the top R0° layer in BLG cannot be determined, it remains speculative whether this band gap is influenced by the observed Umklapp effect of the BLG data, a topic addressed again at the end of this paper.

So far, we can summarize that CVD-grown graphene electronically interacts with the underlying metallic Cu(111) support leading to n-doping and band gap formation in agreement with the literature data.^21,49,51^ The properties change if graphene is synthesized on a Si-loaded Cu foil and Si is segregated to the g-Cu interface by oxygen intercalation at elevated temperature. While the reduction of the n-doping upon oxygen intercalation is already reported in the literature,^49,56^ the data shown in the next paragraph prove the usefulness of the released Si contaminants from the quartz reactor wall as graphene can be synthesized on Cu with the ultrarobust band structure of freestanding graphene at textbook quality.

### Properties of CVD-Grown Graphene on Cu Foils with Intercalated Silica

As already mentioned, graphene may be grown on Si-loaded Cu foil by using SiO released from the quartz tube reactor wall. Subsequent Si segregation is triggered by oxygen intercalation at *T* > 250 °C and leads to the formation of an intercalated silica interface underneath the grown graphene layer.^37^ Figure 3 compiles the LEEM data taken from a Si-loaded Cu sample on which graphene flakes were grown by CVD in a degraded quartz tube that released large amounts of Si. After graphene synthesis, the sample was removed from the reactor and characterized by XPS, showing the absence of Si on the surface. Hence, all of the silicon was dissolved in the Cu bulk. Afterward, the sample was reintroduced in the reactor and exposed to 5075 L oxygen at 950 °C (for details, see the Experimental Section and Supporting Information). This oxygen dosing would burn off the synthesized graphene if grown on a Si-free Cu foil.^37^ However, the synthesized graphene flakes on the highly Si-loaded Cu foil remained intact because all adsorbed and intercalated oxygen atoms were readily converted to silica and did not attack the grown graphene layer.

**Figure 3:**
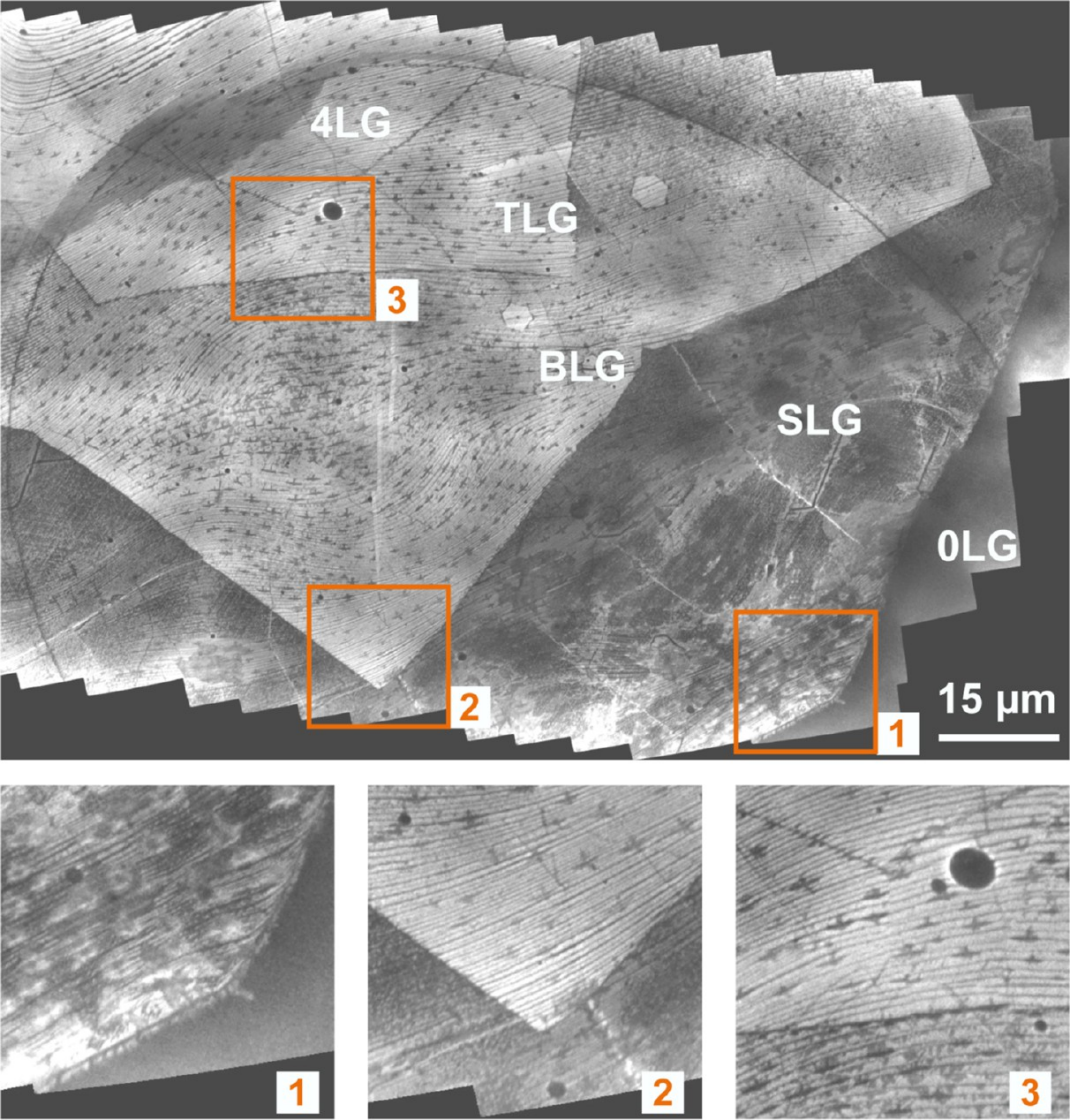
LEEM data taken from a graphene flake which was grown on a Si-loaded Cu foil and exposed to 5075 L oxygen at 950 °C. The sample was imaged at RT after degassing at 160 °C in UHV. Large parts of a graphene flake are displayed with the flake edge in the vicinity of the uncovered Cu foil surface in the lower right corner. The image is constructed by stitching 170 LEEM images (start voltage (STV) = 10 eV at FOV = 15 μm). Magnifications of the orange-framed regions display places where the graphene layer thickness increases stepwise (from SLG to BLG, TLG, and 4LG) and where X-ray photoelectron emission microscopy (XPEEM) data were acquired (see Figures 4 and 5).

Figure 3 shows a large part of a graphene flake in the vicinity of the uncovered Cu foil in the lower right corner. The image was generated by stitching 170 LEEM images (field-of-view (FOV) = 15 μm, STV = 10 V). Three orange boxes indicate areas where the graphene layer thickness increases stepwise and where further LEED and XPEEM data were acquired. Insets display the respective magnified regions below the overview image. Electron reflectivity curves taken in these regions (see Figure S4 of the Supporting Information) prove the single, bi-, tri-, and four-layer thickness of an intact graphene flake. Local LEED data (see Figure S4 of the Supporting Information) corroborate that the imaged graphene flake has the known inverted wedding cake morphology and that the thickness increase follows the stacking of alternating 30°-rotated graphene layers; i.e., the graphene lattice follows an R0°, R30°, R0°... stacking sequence with R0° indicating the orientation of the topmost layer. The diffraction data also confirms that the Cu support surface is faceted with the known staircase morphology of alternating facets.^57,58^

Figure S5 of the Supporting Information compiles the XPEEM data of the flake boundary to the graphene-free Cu surface taken at the indicated area shown in inset “1” of Figure 3. The data shown in Figure S5 provide chemical resolution and reveal the formation of 3-dimensional silicon oxide outside the graphene-covered region. However, SiO_*x*_ is also found underneath SLG as about 3-layer thick, fractally shaped triangular islands (SiO_*x*_-islands) surrounded by a homogeneous surface silica layer on Cu of about bilayer thickness (flat-Cu—see below). Further away from the flake edge toward the inner part of the flake, silica formation at the interface between graphene and the Cu support is still observed, but the fractally shaped silica islands turn into small cross-shaped ones with their long island edge aligned to the stripes of the Cu foil support.

Figure 4 displays the XPEEM data of insets “2” and “3” of Figure 3 which spatially resolve the mentioned effects at the SLG/BLG and BLG/TLG/4LG boundaries of the graphene flake. Each XPEEM image reflects the integrated peak intensity of the C 1s, Cu 3p, Si 2p, and O 1s core-level photoemission, applying the same gray scale for each element with lowest gray scale equaling to zero intensity. As a result, the C 1s images mainly show the stepwise intensity increase with increasing carbon layer thickness. Moreover, the SLG, BLG, TLG, and 4LG regions in the C 1s image appear almost uniform, which proves that graphene is the outermost layer with Si, O, and Cu distributed underneath so that the C 1s photoemission is affected only by the stacking of graphene layers. The rise of the C 1s signal with increasing graphene layer thickness is displayed in the chart below the C 1s XPEEM images with “1” relating to the C 1s intensity of SLG. The blue curve indicates the expected stepwise signal gain with an increase of 30% per additional layer as expected for the signal damping of the emitted photoelectrons with 115 eV kinetic energy through graphene, perfectly matching the C 1s intensity of graphene directly on the Cu substrate (g/flat-Cu).^59,60^ The C 1s intensity extracted from areas containing intercalated cross-shaped SiO_*x*_ islands (g/SiO_*x*_-islands) also almost equals the one obtained from the area surrounding the SiO_*x*_ islands (g/flat-Cu). This holds true especially for SLG-covered areas but deviates to a larger degree with an increase in graphene thickness. Thus, we can conclude that the graphene flake remained intact after oxygen exposure and deviates slightly only where cross-shaped silica islands are observed. Note the straight, bright lines in the C 1s images, which are collapsed wrinkles where the number of stacked carbon layers is locally enhanced and the C 1s emission signal is increased accordingly.

**Figure 4:**
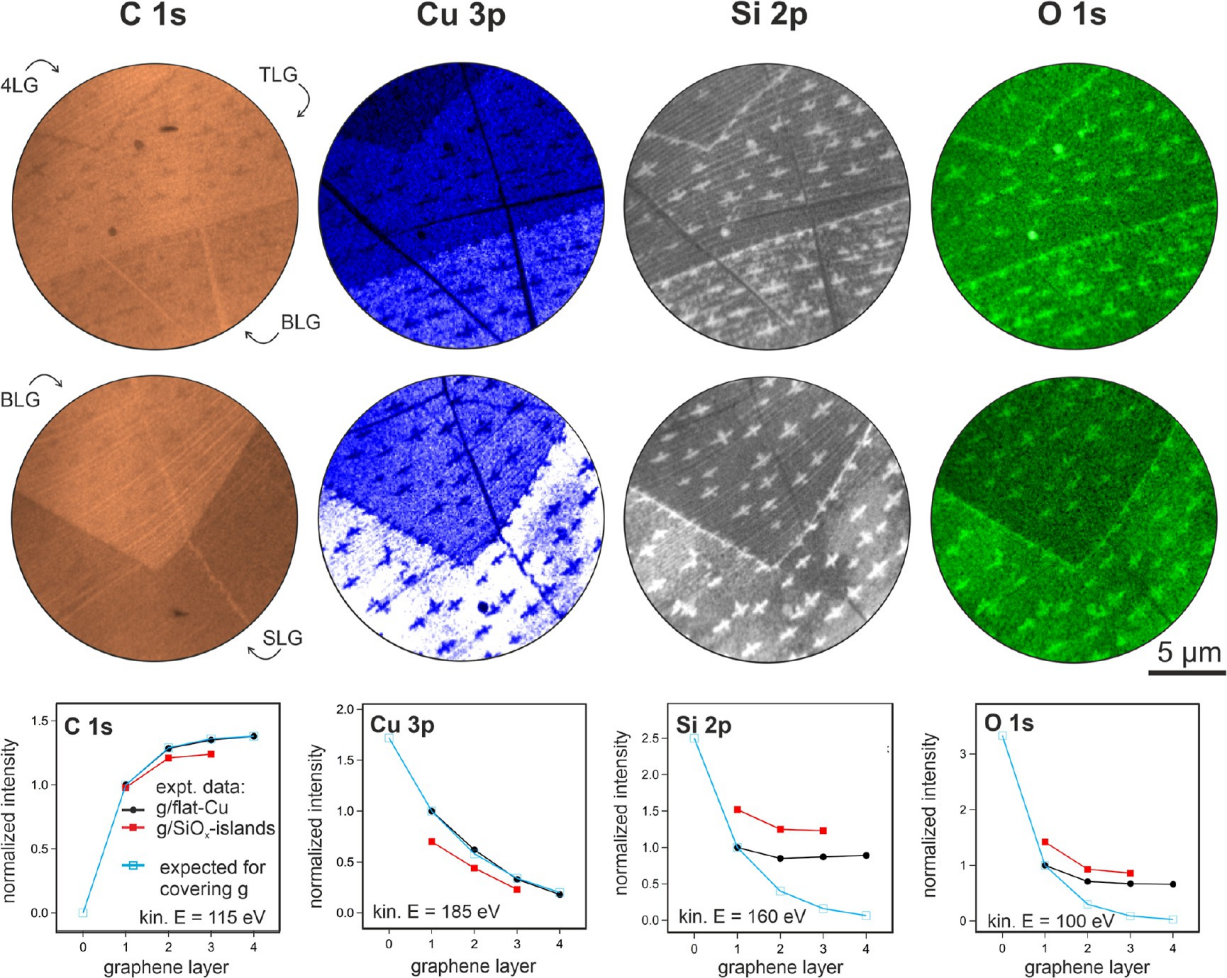
XPEEM images of the graphene flake at the regions “3” (upper row) and “2” (center row) indicated in Figure 3 where the flake thickness increases stepwise from SLG to BLG, TLG, and 4LG. The images are generated by summing the background-corrected photoelectron emission intensity from the O 1s, Si 2p, Cu 3p, and C 1s core levels. For each element, the same color scale is used with lowest intensity corresponding to zero emission intensity. The charts shown in the lowest row below each XPEEM image plot the signal increase or decrease of the respective core-level emission from regions where graphene covers the cross-shaped islands (g/SiO_*x*_-islands) or the surrounding area (g/flat-Cu). The given core-level peak intensities are scaled to their respective intensities obtained from the SLG-covered (g/flat-Cu) area and are plotted versus the thickness of the covering graphene. The regular C 1s increase with subsequent carbon layers together with the Cu 3p decrease proves a covering graphene film on top of the supporting Cu foil. The irregular intensity profile of the O 1s and Si 2p photoelectron emission indicates that the distribution of the intercalated silica changes with increasing graphene thickness in a way that the photoemission signal damping through the covering graphene layers is essentially counterbalanced by an enhanced Si 2p and O 1s emission yield under thick graphene (see text). Note that the intensities extracted from the regions covered by cross-shaped silica islands (g-SiO_*x*_-islands) differ slightly with respect to the ones from the surrounding area (g/flat-Cu), indicating that the cross-shaped islands are large 3-dimensional objects which might partly cut through the covering graphene layer. Photon energies: C 1s data: 400 eV, Cu 3p and Si 2p data: 260 eV, and O 1s data: 630 eV.

The Cu 3p data set reflects the photoemission signal of the support foil, which hosts the graphene flake and the intercalated silica. Thus, each covering layer further attenuates the Cu 3p photoemission signal. In the case that the intercalated cross-shaped silica islands (SiO_*x*_-islands) and the silica on the surrounding Cu foil surface (flat-Cu) are equally distributed independent of the thickness of the covering graphene flake, the Cu 3p signal attenuation of both surface phases should exclusively depend on the number of covering carbon layers of the graphene flake. The blue curve in the Cu 3p chart displays the expected relative Cu 3p signal attenuation per covering graphene layer extracting a 40% signal loss per layer. The blue curve perfectly matches the experimental data, although a slightly larger damping of about 50% signal loss per graphene layer would be expected at the photoelectron kinetic energy of 185 eV.^59,60^ However, the silica concentration underneath graphene might vary, which might have a compensating effect accounting for the slight deviation of the signal attenuation. The Cu 3p signal damping by the cross-shaped silica islands is well seen in the chart and also spatially resolved in the Cu 3p images where the cross-shaped silica islands appear as dark patches. Finally, collapsed wrinkles in the graphene flake are resolved in the Cu 3p images as straight dark lines corresponding to the expected signal damping due to an increased graphene layer thickness at positions where graphene is folded on top of each other.

Consistent with this assignment, the intercalation of silica is verified when considering the Si 2p and O 1s XPEEM images in which the cross-shaped silica islands (SiO_*x*_-islands) appear bright. As already mentioned, intercalated silica is also found on the (g-flat-Cu) region and, especially on the SLG part of the graphene flake, the intercalated silica is nonhomogeneously distributed as observed by the lateral Cu 3p intensity variation which appears at inversed contrast in the Si 2p and O 1s images. In accordance with the formation of silica, Si 2p and O 1s XPEEM images deliver equal contrast. However, the O 1s data set suffers from a low signal-to-noise ratio due to the low O 1s cross section and the low photon intensity at the used photon energy of 630 eV so that the Si 2p images provide more reliable information. As outlined in the Supporting Information with the help of Figure S5, the average silica loading under SLG amounts to a bilayer. The almost equal C 1s photoemission yield acquired from the (g/SiO_*x*_-islands)- and (g/flat-Cu) regions in this area indicates an intact covering SLG film.

Below thicker graphene layers, the Si 2p and O 1s photoemission signals drop as expected for intercalated silica. However, quantitatively, deviations arise from the screening as a function of the graphene thickness. This is best seen in the Si 2p and O 1s intensity charts plotted below the XPEEM images in Figure 4. Here, the blue curve plots the expected signal drop of the photoelectron emission yield at the given kinetic energy if a homogeneous silica film was completely covered by graphene. The deviations from the expected curves are obvious and indicate that there must exist a compensating effect regarding the Si 2p and O 1s photoemission yields counterbalancing the signal damping of the covering graphene layers. The most likely explanation is that the intercalated silica does not homogeneously cover the Cu interface and has heterogeneity dependent on the thickness of the graphene overlayer. This is verified in Figure 5, which displays the Si 2p/Cu 3p ratio image computed from the BLG, TLG, and 4LG data set of Figure 4. First of all, the ratio image clearly resolves the cross-shaped silica islands (g/SiO_*x*_-islands) but also reveals the striped contrast of the (g/flat-Cu) region. As discussed with the help of Figure 3, the stripes emerge from the staircase morphology of the Cu support foil with alternating differently inclined facets. Since the photoelectron yield at perpendicular emission differs from the one at more grazing emission,^61^ also XPEEM data taken from the Cu foil contain geometric contrast due to the alternation of differently inclined facets. However, this contrast mechanism affects both the Cu 3p and the Si 2p image to almost equal extent due to the similar kinetic energy (*E*_kin_) of the emitted photoelectrons (Si 2p: *E*_kin_ = 160 eV and Cu 3p *E*_kin_ = 185 eV). As a result, the geometric contrast is removed from the ratio image so that the gray level contrast reflects the different silica loading on the alternating Cu facets.

**Figure 5:**
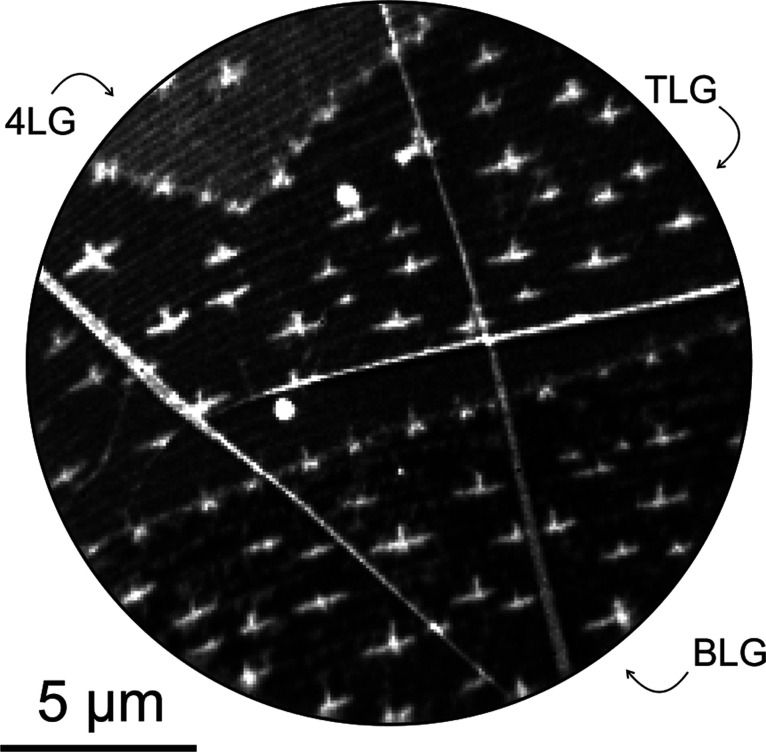
Si 2p/Cu 3p intensity ratio image computed from the BLG/TLG/4LG data set of Figure 4. The ratio image clearly resolves the cross-shaped silica islands and the stripe appearance of the underlying Cu foil. Geometric contrast caused by variation of the photoelectron yield due to emission from differently inclined surface facets cancels in the photoemission ratio image. Thus, the image contrast reflects the different silica loading of the alternating Cu foil facets. Note that also the damping effect of the covering graphene is removed in the image. Close inspection shows that not only large, cross-shaped islands decorate the alternating facets but also the decoration by smaller silica islands is observed in the BLG region. The small islands appear very faint under TLG and are not spatially resolved at all under the 4LG-covered flake region. We suggest that the decorating silica islands shrink in height but wet an increased Cu area when being covered by thicker graphene layers (see text). The result is the increased stripe contrast level from BLG to 4LG in the Si 2p/Cu 3p image. The effect is counterbalancing the signal damping by the covering graphene layer in Figure 4 and explains the observed irregular Si 2p and O 1s emission scaling.

Close inspection of the ratio image in Figure 5 shows that apart from the well-visible large cross-shaped islands, also smaller silica islands are laterally resolved to decorate the Cu facets. The result is the stripe contrast of the (g/flat-Cu) region of the graphene flake. At the BLG region, the decorating silica is imaged as well-separated islands that are barely resolved under TLG and finally turn into uniform stripes under the 4LG covered part of the flake. Thus, we conclude that the silica decoration takes place at the minority facet type of the Cu support foil with silica islands shrinking in size with increasing graphene overlayer thickness. The distribution of the same silica amount coalesced in few and high islands into many small ones that wet a larger Cu facet area will lead to an enhancement of the net Si 2p intensity, and in fact, the gradual stripe brightness increases from BLG to TLG and 4LG as well resolved in Figure 5. Note that the observed contrast increase in the Si 2p/Cu 3p image does not depend on the thickness of the graphene flake because in the ratio image not only the geometric contrast cancels out but also the damping effect of the covering layers.

The data also explain the findings of Figure 4 with a regular C 1s and Cu 3p intensity scaling and the irregular Si 2p and O 1s photoemission intensity variation from the TLG and 4LG region of the graphene flake: the redistribution of the intercalated silica islands into smaller islands under thicker graphene leads to the enhancement of the average Si 2p and O 1s intensity counterbalancing the signal damping of the covering graphene layer. Instead, since the silica decoration takes place at the minority facet of the Cu foil, the Cu 3p signal is only weakly affected and follows the expected intensity decrease with increasing graphene layer thickness.

The driving force affecting the postulated silica island distribution might be the correlation between defects in the graphene layer and the nucleation sites of the intercalated silica. Oxygen atoms adsorbing on a thin flake directly reach the Cu surface at the defect site in the graphene layer where they immediately attract the dissolved silicon, nucleating the silica island, which grows as long as the oxygen reaches the interface in the vicinity of the island. On a thick graphene layer, instead, adsorbing oxygen atoms diffuse though the graphene layers until reaching the interface where they nucleate silica islands at random sites and low growth rate, which would explain an island distribution of small decorating silica islands. There exists also an energetic argument favoring the redistribution of large silica islands into smaller ones under thick graphene because taller silica islands locally bend the covering graphene to a larger extent in comparison to the equal amount of silica distributed in many small, intercalated islands. Since graphene turns stiff with increasing thickness, the latter process is preferred.

Even though other configurations of intercalated silica may be considered, such models seem considerably less likely to be consistent with the data of Figures 4 and 5 at the same time. This issue is discussed in the Supporting Information where details regarding the computation of Figure 5 are also given (see Figure S6). Summing up, the XPEEM data clearly provide evidence of Si segregation during oxygen exposure in the CVD reactor, which leads to the formation of intercalated silica. The result is an electronically completely decoupled graphene layer on the Cu support as discussed in the following.

Figure 6 displays momentum plots acquired at the indicated binding energies from the silica-intercalated SLG, BLG, and TLG regions of the graphene flake imaged in Figures 3, 4, and 5, where 30°-rotated graphene layers are stacked one on another (Videos S3, S4, and S5 of the full data sets are given in the Supporting Information). The red lines in the momentum plot at −1.65 eV binding energy display the intensity cuts along *K* ^′^-Γ-*K* and along the perpendicular direction at the *K*-point. The extracted data reflects the electronic band structure of the 2-dimensional graphene system and is shown in Figure 7. Sketches to the right indicate the differently aligned carbon layers in SLG, BLG, and TLG.

**Figure 6:**
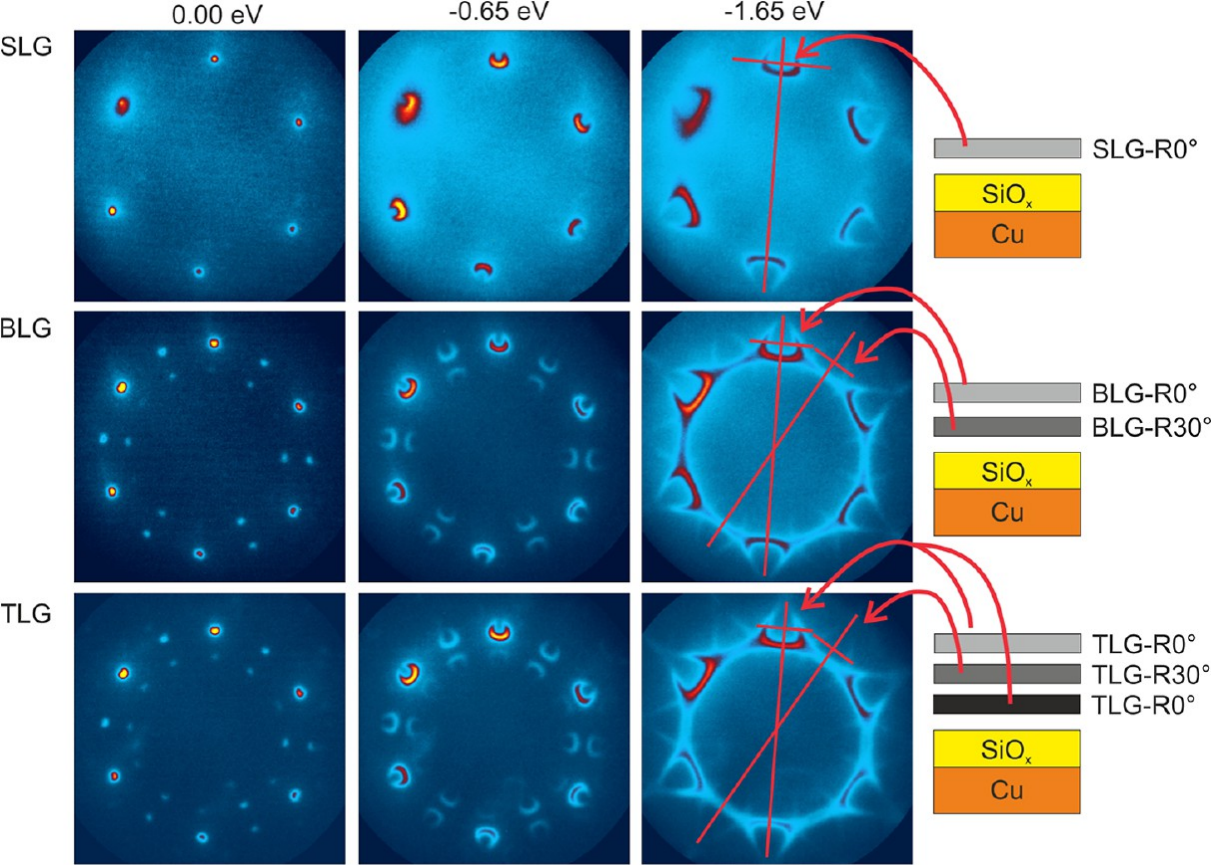
Left: Photoelectron momentum plots taken at the indicated binding energies from SLG, BLG, and TLG regions of the g/SiO_*x*_/Cu sample. Replica spots caused by the photoemission from the R30° layer in BLG and TLG are observed, while replicas from the R0° layer appear only in TLG. Red lines indicate cuts through the *k*-space from which band structure data were extracted (see Figure 7). Right: Sketch of the stacked graphene layers that contribute to the respective photoelectron emission features of the momentum plots (Videos S3, S4, and S5 of the data sets are given in the Supporting Information). Photon energy 46 eV.

**Figure 7:**
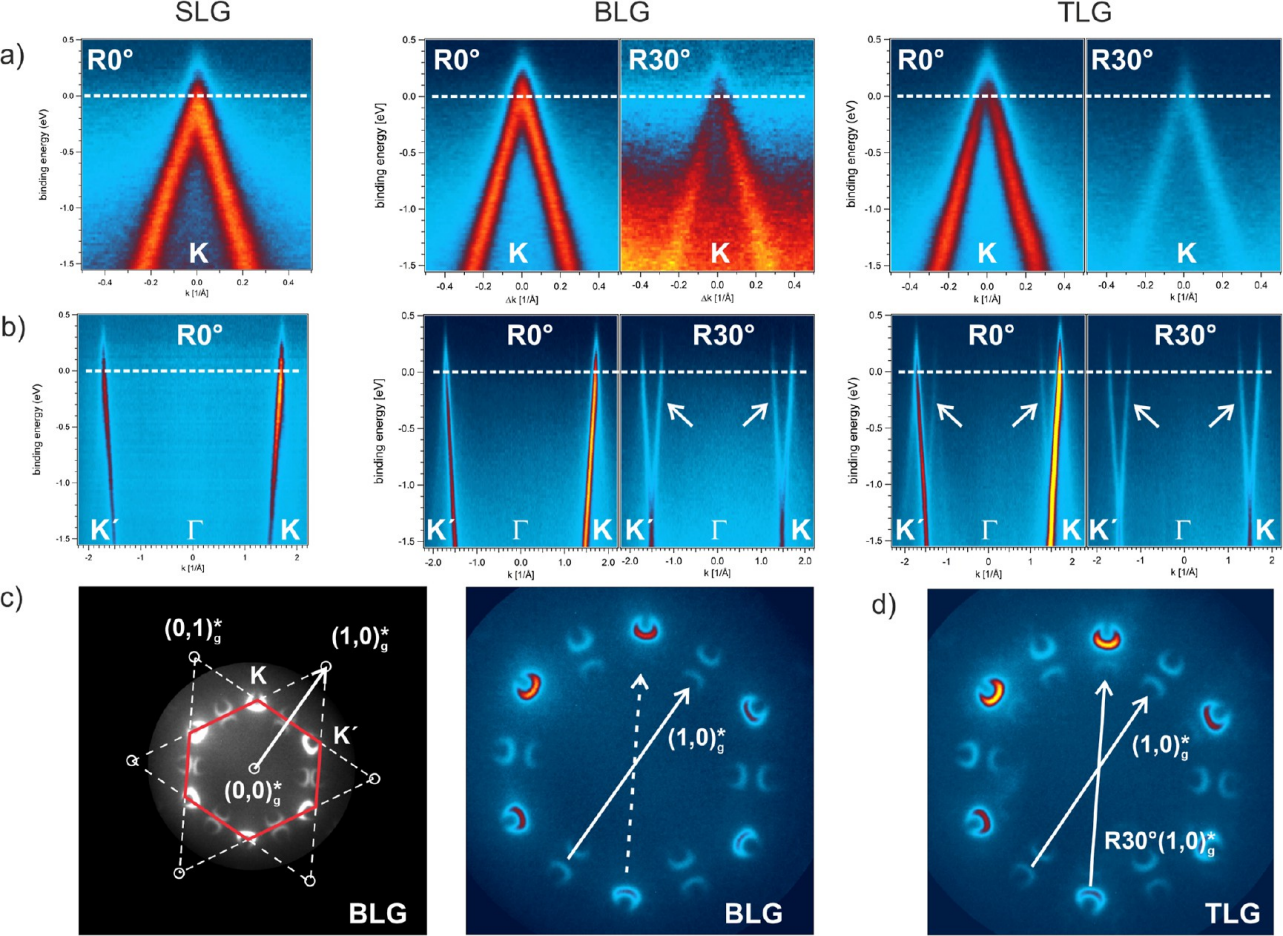
Band structure data along the ***K*** ^′^-Γ-***K*** and the perpendicular direction at the ***K***-point extracted from the momentum plots of Figure 6. (a) Band structure at the ***K***-point of SLG, BLG, and TLG. The strictly linear energy dispersion of the band and the absence of any energy gap are readily observed together with the band crossing at 0.00 eV. The Dirac point located at the Fermi edge proves the electronic band structure of undoped, free-standing graphene for each carbon layer of SLG, BLG, and TLG. (b) Band structure along the ***K*** ^′^-Γ-***K*** direction also indicates the linear energy dispersion of the π-band with a Fermi velocity of (1.1 ± 0.1)·10^6^ m/s in each layer of SLG, BLG, and TLG. The photoelectron emission from the R30° layer of BLG displays replica bands that do not appear for the R0°-rotated graphene lattice. In TLG, replica bands are found for photoelectrons originating from both the R0°- and the R30°-rotated graphene layer. (c) Left panel: BLG momentum plot of the first Brillouin zone embedded in the reciprocal graphene R0° lattice. Center panel: BLG momentum plot with one reciprocal lattice vector (white arrow) indicating the momentum transfer during electron scattering, which leads to one of the replicas: a photoelectron is emitted from the bottom R30° layer in BLG and scatters in the topmost R0° graphene layer on its way to the electron detection system. (d) Right panel: since a R0°-rotated graphene layer is stacked below the R30° layer in the TLG region of the flake, the replica formation exists for photoelectrons originating from the R0° and the R30° reciprocal graphene lattice. The latter is not observed for BLG as indicated by the dashed arrow in (c).

First, the momentum plots acquired at a 0.00 eV binding energy from SLG, BLG, and TLG display point-like photoelectron emission at the *K*-point, indicating the presence of the Dirac point at the Fermi level of the electronic system. This assignment will be confirmed further below when the band structure is discussed with the help of Figure 7. The second finding is the observation of replica spots in the momentum plots of BLG and TLG. In the photoelectron emission data from BLG, replica spots originate from the R30° but not from the R0° lattice of the two stacked graphene layers. Instead, on TLG, replicas in *k*-space are observed due to photoelectron emission from both rotated lattices.

Figure 7a,b plots the extracted photoelectron emission intensity versus binding energy along the two reciprocal space directions indicated in the momentum plots of Figure 6. The respective cuts at the *K*-point (Figure 7a) clearly display the linear band dispersion of two bands crossing the *K*-point when reaching the Fermi level. The data confirm undoped graphene for every carbon layer in SLG, BLG, and TLG with the Dirac point at 0.00 eV binding energy located at the *K*-point. No indication of a band gap appears as the band proceeds strictly linearly up to the Fermi level. The band structure along the *Ḱ*-Γ-*K* direction is plotted in Figure 7b and also proves the linear dispersion of the π-band with a Fermi velocity *v*_F_ of (1.1 ± 0.1) m/s independent of the nature of the carbon layer in SLG, BLG, and TLG. Note that an increase of *v*_F_ when approaching the Dirac point is not observed in contrast to the reported increase for graphene transferred on SiO_*x*_ or other dielectric supports.^62^ Thus, in our case, the intercalated silica does not influence the electronic system, and the properties of free-standing undoped SLG are met for each covering graphene layer, no matter whether it is present in SLG or stacked in BLG or TLG.

The data also show the occurrence of replica bands in the photoelectron emission data from the R30° layer in BLG and TLG, which (obviously) are not found for the R0° carbon lattice in SLG. In addition, replica formation from the R0° lattice appears in TLG. The scattering event responsible for replica band formation can be discussed with the help of Figure 7c. The left panel shows the momentum plot of BLG embedded in the sketch of the reciprocal lattice of unrotated graphene (R0°). In the right panel, the translated (1,0)*g reciprocal lattice vector is sketched as a white arrow, which induces the replica formation at the *K*-point of the R30° lattice in BLG. The scattering event takes place if photoelectrons are emitted from the 30°-rotated second layer in BLG or TLG and pass the unrotated covering R0° carbon layer on their way to the detection system. While penetrating the nonrotated layer, they may scatter and pick up a reciprocal lattice vector, leading to the replica formation of the R30° lattice-related photoemission data. Since an equivalent process is possible for photoelectrons ejected from the R0° lattice in the lowest carbon layer of TLG, replica formation is also observed for the R0° lattice-related photoelectron emission in TLG as sketched in Figure 7d.

Note that replica formation of the R0° lattice is completely absent in decoupled BLG (see the dashed arrow in Figure 7c), while it appeared at a low intensity in BLG on metallic Cu(111) (see Figure 1). It was already discussed that replica formation in the band structure of the topmost graphene layer in BLG requires photoelectron emission toward the bulk of the supported graphene followed by backscattering in or below the second layer. On the way back toward the surface, the scattering at the 30°-rotated graphene lattice would initiate the required Umklapp process. In any case, the observation of Umklapp processes on decoupled BLG and TLG without electronic band gap formation clearly proves that exclusively excited photoelectrons can pick up reciprocal lattice vectors on their way to the electron detection system, while the remaining electrons in each graphene layer do not interact at all, as this would lead to the formation of a band gap. Since the stacking of R30°-rotated graphene layers does not lead to the formation of a commensurate structure,^63^ this property might support the complete electronic separation of each stacked carbon layer from one to another. Note that this property leads to the fact that Raman spectra taken from R30°-stacked multilayer graphene equal the ones of SLG, which has led to the erroneous assignment of suspended graphene membranes to SLG, as noted in the literature.^58^

## Conclusion

In our study, we have shown that graphene can be CVD grown on Cu foils with the ideal electronic band structure of graphene on metallic Cu(111), the n-doping of SLG by −0.4 eV, and the band gap formation of about 0.3 eV. With increasing graphene layer thickness, the n-doping decreases. The observed downward shift of the graphene energy bands is consistent with the electron transfer from Cu to graphene because the corresponding upward energy shift of the Cu(111) surface state is also observed, in agreement with the literature data.^21^ If instead, a protocol described in the literature is followed where CVD-grown graphene on Si-loaded Cu is subsequently exposed to oxygen at elevated temperature, Si segregation toward the graphene–Cu interface and its conversion to intercalated silica is induced.^37^ As a result, the CVD-grown graphene electronically decouples, and the textbook-like band structure of free-standing graphene emerges. Our data also reveal that the stacking of 30°-rotated lattices in bi- and trilayer thick graphene leads to electronically noninteracting layers. However, photoelectrons released from one lattice may scatter when penetrating a rotated graphene lattice on their way to the analyzing system and pick up the momentum of a reciprocal lattice vector. As a result, the ARPES data show replica bands due to Umklapp processes, but no energy gap emerges.

## Experimental Section

Two samples were synthesized on a 25 μm-thick Cu foil (Alpha Aesar 99,8% purity) by CVD in a home-built quartz tube reactor, described elsewhere.^64,65^ The synthesis protocols consisted of a Cu foil pretreatment inducing its recrystallization followed by the actual CVD growth of graphene (growth conditions summarized in the Supporting Information). Both samples were synthesized on Si-loaded Cu foil and brought into contact with air during removal from the CVD reactor. However, the first sample (shown in Figures 1 and 2) was gently degassed in the measurement chamber after air contact so that almost all intercalated oxygen was desorbed before reaching the temperature where Si becomes mobile, and silica conversion would set in. Since in this case the Si remains dissolved in the Cu bulk, the investigated sample has the properties of CVD-grown graphene on metallic copper.^37^ As outlined in the Supporting Information, the characterized graphene resided on an almost adsorbate-free Cu(111) grain of the Cu foil.

In contrast to the first sample, the second CVD-grown graphene sample (data of Figures 3, 4, 5, 6, and 7) was reintroduced into the reactor where it was annealed up to 950 °C in 1 mbar H_2_ and then exposed to 5075 L O_2_ to induce Si segregation and conversion into intercalated silica. Note that such oxygen exposure would burn off graphene on Si-free Cu foils. Suitable parameters for reactor treatment enabling the wanted silica intercalation and graphene decoupling are compiled in the Supporting Information, together with optical microscopy and Raman spectroscopy data.

After synthesis, both samples were removed from the reactor, stored in a sample container, and transported to the Elettra synchrotron light facility, where they were introduced into the measurement chamber and gently degassed, which removed loosely bound adsorbates but did not affect the intercalated silica layer. The samples were characterized using the spectroscopic photoelectron and low energy electron microscopy instrument at the nanospectroscopy beamline of the Elettra synchrotron light facility which is described elsewhere.^66,67^ The data presented in this study used various imaging modes. (A) Upon irradiation with electrons: LEEM: energy-filtered imaging of the real space with elastically reflected electrons at the given STV. The images shown in our study use the reflected electrons from the (0,0) diffraction spot (bright-field imaging). LEEM- I/V: reflected electron intensity (I) curves of the (0,0) spot versus the given STV as extracted from selected areas of bright-field LEEM images. Area-selective low-energy electron diffraction (μ-LEED) is energy-filtered reciprocal space imaging of the reflected electrons acquired from a 2 μm wide spot of the sample at the given STV. (B) Upon irradiation with photons: XPEEM: energy-filtered real space imaging by acquiring the photoelectrons released from the indicated core levels. Angular resolved photoelectron spectroscopy (ARPES): energy-filtered reciprocal space image (momentum plots) of the emitted photoelectrons acquired from a **2** μm wide spot of the sample at the given STV. The different data acquisition modes are further outlined in refs 37 and 66 The ARPES data sets were taken by acquiring momentum plots in energy steps of 25 meV. The binding energy scales of the data sets recorded from sample 1 and sample 2 were determined by locating the position of the Fermi edge. This was achieved by fitting a Fermi function to the photoelectron emission intensity versus the kinetic electron energy by averaging over many *k*-space points along the *K* ^′^-Γ-*K* direction. The resulting fits of the respective data sets showed energy resolutions of 0.30 eV (sample 1) and 0.40 eV (sample 2), which allowed the location of the Dirac point with a precision of about 0.05 eV.

## Supplementary Material













## Notes

ARPES data sets acquired from SLG on a (111) grain of the metallic Cu foil (MP4)
ARPES data sets acquired from BLG on a (111) grain of the metallic Cu foil (MP4)
ARPES data sets acquired from SLG on a Cu foil support with intercalated silica (MP4)
ARPES data sets acquired from BLG on a Cu foil support with intercalated silica (MP4)
ARPES data sets acquired from TLG on a Cu foil support with intercalated (MP4)
Experimental details and data treatment including additional LEEM, XPEEM data, as well as optical and secondary electron microscopy and Raman spectroscopy data (PDF)

## ∥

Interdisciplinary Nanoscience Center, Aarhus University, Gustav Wieds Vej 14, 8000 Aarhus C, Denmark

## ⊥

Helmholtz-Zentrum Berlin für Materialien and Energie GmbH, BESSY II Albert-Einstein-Straße 1512489Berlin, Germany.

## #

CNR—Istituto Officina dei Materiali (IOM), S.S. 14—km 163,5 in Area Science Park, 34149 Basovizza, Trieste, Italy.
