# Supplementary material for: Making Use of Si Contaminants during Chemical Vapor Deposition of Graphene on Cu: Synthesis of a Stable Material with the Textbook-like Band Structure of Free-Standing Graphene
Source: ACS Appl Mater Interfaces. 2025 Jul 2;17(28):40937–50. doi: 10.1021/acsami.5c06939 (PMC12278212; doi:10.1021/acsami.5c06939)
Supplement: Supplementary file 6 [file am5c06939_si_006.pdf]

Supporting information

Making use of Si contaminants during chemical vapor deposition of graphene on Cu: Synthesis of a stable material with the textbook-like band structure of free-standing graphene

Tim Kratky[1], Jürgen Kraus[1], Paul M. Leidinger[1,%], Patrick Zeller[1,#], Francesca Genuzio[2,$], Alessandro Sala[2,$], Tevfik Onur Menteş[2], Andrea Locatelli[2] and Sebastian Günther[1,*]

[1]Technical University of Munich (TUM), Department of Chemistry, Physical Chemistry with Focus on Catalysis, Lichtenbergstr 4, Garching 85748, Germany, Catalysis Research Center, Ernst-Otto-Fischer-Str. 1, 85748 Garching, Germany

[2]Elettra-Sincrotrone Trieste S.C.p.A., S.S. 14 - km 163,5 in Area Science Park, 34149 Basovizza, Trieste, Italy

[%]present address: Interdisciplinary Nanoscience Center, Aarhus University, Gustav Wieds Vej 14, 8000 Aarhus C, Denmark

[#]present address: Helmholtz-Zentrum Berlin für Materialien und Energie GmbH, BESSY II Albert-Einstein-Straße 15, 12489 Berlin, Germany

[$]present address: CNR - Istituto Officina dei Materiali (IOM), S.S. 14 - km 163,5 in Area Science Park, 34149 Basovizza, Trieste, Italy

*(*Corresponding author: S. Günther, e-mail: sebastian.guenther@tum.de)*

First sample - synthesis protocol and structure of CVD grown graphene on metallic Cu:

The sample was annealed to 950 °C in an Ar atmosphere of 12 mbar to which the oxygen partial pressure of 0.8x10(-3) mbar was added. After 75 min the temperature was raised to 1045 °C and the sample was kept at this temperature for further 60 min. Afterwards the sample was exposed to 300 mbar hydrogen for 160 min. The sudden change from oxidative to a reductive atmosphere induces the conversion of the polycrystalline into a (111) textured Cu foil.[1] At the same time, the quartz tube releases SiO so that the Cu foil is Si loaded. The SiO release is triggered at elevated pressure and temperature ($p(H_2) > 100$ mbar, t > 1 h, T > 1000 °C) in a hot wall quartz tube reactor. The reductive conditions induce the release of SiO from the quartz wall, which is transported to the Cu foil where it is reduced and dissolved in the Cu bulk.[2] The actual CVD synthesis took place at T = 1045°C, $p(H_2) = 20$ mbar, $w = p(H_2) / p(CH_4) = 1000$ for a growth time of 20 min. During CVD growth the Si remains highly dissolved in the Cu bulk so that it does not influence the quality of the grown graphene.[2]

After synthesis, the sample was carried to the nanospectroscopy beamline, inserted into the preparation chamber of the SPELEEM instrument and gently degassed ramping the temperature slowly up to 250 °C so that all intercalated adsorbates were removed without dragging Si to the surface avoiding silica formation. The silicon and oxygen coverage on this sample was determined by XPEEM as being about 10 times smaller than the one found on the second sample (see Figure S5: here the respective O 1s and Si 2p peaks of the first sample would appear almost as straight lines). The sample

was then transferred to the microscope chamber, safely annealed up to 500 °C and cooled down to room temperature for further characterization.

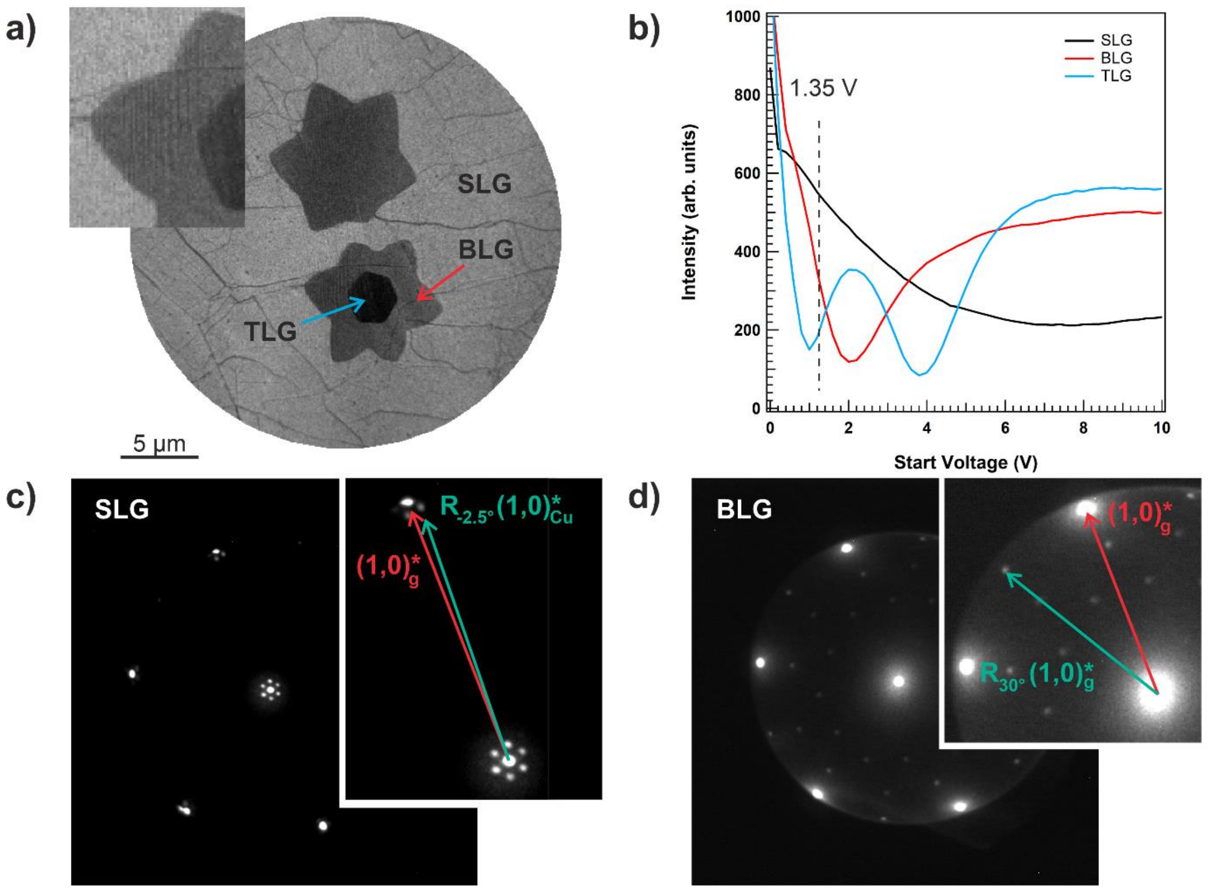

Figure S1. a) LEEM image at STV = 1.35 V, FOV = 30 µm, SLG, BLG and TLG is imaged at stepwise decreasing brightness. Dark irregular lines are wrinkles in the graphene layer. Inset: left part of the lower island displays parallel vertical stripes reflecting the staircase morphology of a faceted Cu foil consisting of two inclined surfaces with Cu(111) as the majority fraction of the foil (see below). b) LEEM I(V) curves recorded from the SLG, BLG and TLG region of the imaged area reflecting the typical intensity oscillations of SLG, BLG and TLG. The dashed line indicates the STV at which the image of Figure S1a was taken. c) LEED pattern acquired from SLG at 45 eV. The diffraction pattern shows the spots of the graphene lattice (red) and the underlying Cu(111) support (blue). The Cu(111) lattice is slightly rotated by about – 2.5 ° with respect to graphene. Due to the almost identical lattice constants of both lattices, moiré spots appear in the close vicinity of the (0,0) and first order diffraction spots. d) LEED pattern taken from BLG at 45 eV. The intense (1,0)*g spot (red) and the weak R30°(1,0)*g spot (green) identify a BLG island with inverted wedding cake morphology where the second, 30° rotated graphene layer resides below the SLG layer. Diffraction spots caused by the Cu(111) support underneath BLG are not resolved so that the pattern displays the moiré spots originating from the two rotated graphene lattices only.

Figure S1a displays the real space LEEM image of the sample at this stage resolving two star-shaped islands in the field of view (FOV). The upper one is bilayer thick graphene (BLG) and the lower one is a BLG island with a hexagonally-shaped three layer thick graphene (TLG) core. The SLG, BLG and TLG covered areas appear with stepwise decreasing brightness in the brightfield LEEM image acquired at a

start voltage (STV) of 1.35 V. Dark irregular lines indicate wrinkles in the SLG graphene film. Extracting the intensity of the SLG, BLG and TLG region of the brightfield LEEM image as a function of the STV leads to the electron reflectivity curves of the specular (0,0) beam, called the LEEM I(V) curves which are compiled in Figure S1b. The prominent reflectivity oscillations between 0 and 8 eV identify the graphene layer thicknesses as the ones of SLG, BLG and TLG.[3, 4] The dotted line at STV = 1.35 V helps to identify the electron reflectivity from the SLG, BLG and TLG region which accounts for the contrast of the LEEM image shown in Figure S1a. Figure S1c and S1d display electron diffraction data acquired from SLG and BLG taken at about 45 eV. The diffraction spots obtained from SLG display the moiré pattern generated by the graphene lattice and the about 2.5° clockwise-rotated lattice of the underlying Cu(111) support surface.[5] In the inset of Figure S1c, the R0°-(1,0)*g vector is indicated (red) together with the R-2.5°(1,0)*Cu vector of the Cu(111) support (green). Note that the (111)-textured Cu foil underneath graphene typically follows a staircase morphology of differently inclined surfaces where one represents Cu(111) and the other one a faceted surface which compensates for the local inclination of the Cu foil surface similar to the morphology observed for graphene on (100)-textured Cu foils.[4, 6] Areas with such a morphology appear as parallel stripes in LEEM images such as the ones visible in the left part of the BLG island (see inset). However, the LEED and the ARPES data (shown in Figure 1 and 2 of the main text) were collected in areas without a striped appearance, i.e., at areas where the Cu(111) surface is the majority plane, so that these data exclusively refer to graphene ontop of Cu(111) only.

Similar to the SLG region, the diffraction pattern obtained from the BLG island displays the interaction of two stacked layers only. In this case, the outermost layer has the same orientation as the graphene lattice in the SLG region, which is proven by the same alignment of the intense first-order diffraction spots in Figure S1d. The (1,0)*g vector is sketched (red) in the inset of the figure. Additional sharp, but faint diffraction spots appear which originate from the moiré caused by the 30 ° rotated graphene layer below the outermost unrotated graphene lattice. In the inset, the R30°(1,0)*g vector is sketched in green. The faint intensity of the 30° rotated diffraction spot proves electron diffraction from deeper layers in agreement with the known inverted wedding cake morphology of BLG.[7] Note that no diffraction spots of the underlying Cu(111) lattice are observed. This can be understood since the electron diffraction peaks from the second carbon layer are extremely faint and thus most likely occur due to double scattering only.[5, 8] Also note, that the determined 30° rotation angle of both layers in BLG is based on the location of the first order diffraction spots with an error bar of about 1°.

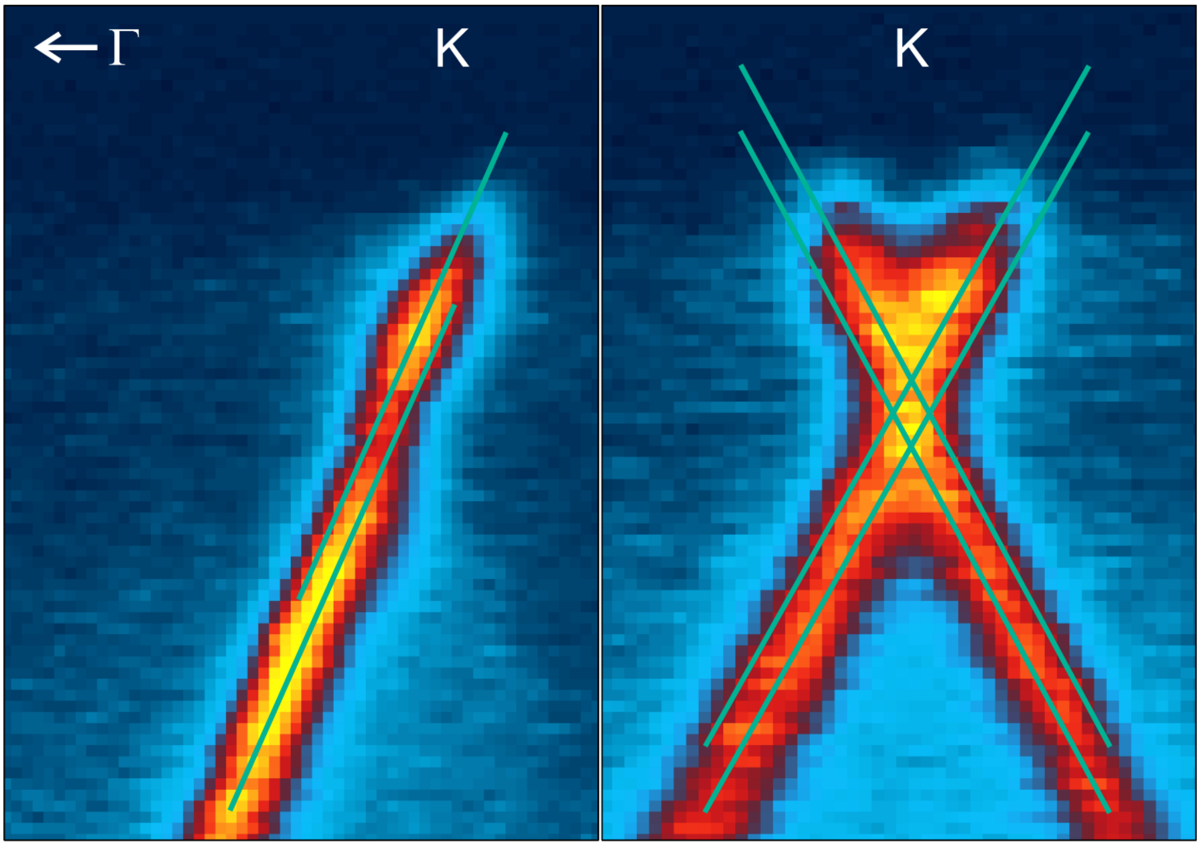

Figure S2. Magnified regions of the SLG /Cu ARPES Data shown in Figure 2 of the main text evidencing the presence of an energy gap. The linear band dispersion along the $\Gamma - K -$ direction is displayed indicated in the left panel, the one at the K-point along the perpendicular direction is shown in the right panel. Green lines clearly indicate the vertical displacement of the energy bands above and below the energy gap.

The energy resolution of the performed experiment amounts to 0.30 eV as determined by a fit of the Fermi edge. As a result, the energy gap of SLG on Cu(111) of the same size (0.30 eV) smears out. However, the splitting into vertically shifted bands at the K-point clearly indicates the presence of an energy gap as indicated in Figure S2.

Second sample - synthesis protocol and structure of CVD grown graphene on metallic Cu with intercalated silica:

The graphene synthesis on the second sample was again performed by CVD on a Si-loaded Cu foil. Here, the Si loading was realized synthesizing the graphene layer in a degraded quartz reactor tube that was exposed many times to hydrogen at elevated pressure and temperature and releases SiO at much milder conditions in accordance with the reported degradation of the quartz reactors with time.[9]

The synthesis protocol consisted of the temperature ramping to 950 °C in 1 mbar hydrogen within 60 min, followed by a 60 min oxygen exposure in 1 mbar Ar to which the oxygen partial pressure of 7.5x10(-6) mbar was added. After this treatment, the temperature was raised to 1075 °C in the Ar/O_2 mixture for a further 15 min followed by the actual CVD growth step by changing the reactive atmosphere to a mixture of $p(H_2):p(CH_4) = 1000$ at a total pressure of 55 mbar for 120 min.

After graphene synthesis, the sample was removed from the reactor and characterized by XPS showing the absence of Si on the surface proving that all silicon was dissolved in Cu bulk. Afterwards, the sample was re-introduced in the reactor, heated in 1 mbar hydrogen up to 950 °C and then exposed to the partial oxygen pressure of $p(O_2) = 7.5 \times 10(-6)$ mbar, dissolved in an Ar carrier atmosphere of 1 mbar for 15 min. The total dosing of 5075 L oxygen would burn off the synthesized graphene if it had been grown on a Si-free Cu foil. However, on the highly Si-loaded Cu foil, the synthesized graphene flakes remained intact because the oxygen atoms adsorbing beside or intercalating underneath the graphene flake were readily converted into silica. Optical microscopy (OM) and secondary electron microscopy (SEM) proved the presence of graphene flakes on the Cu foil as shown in Figure S3.

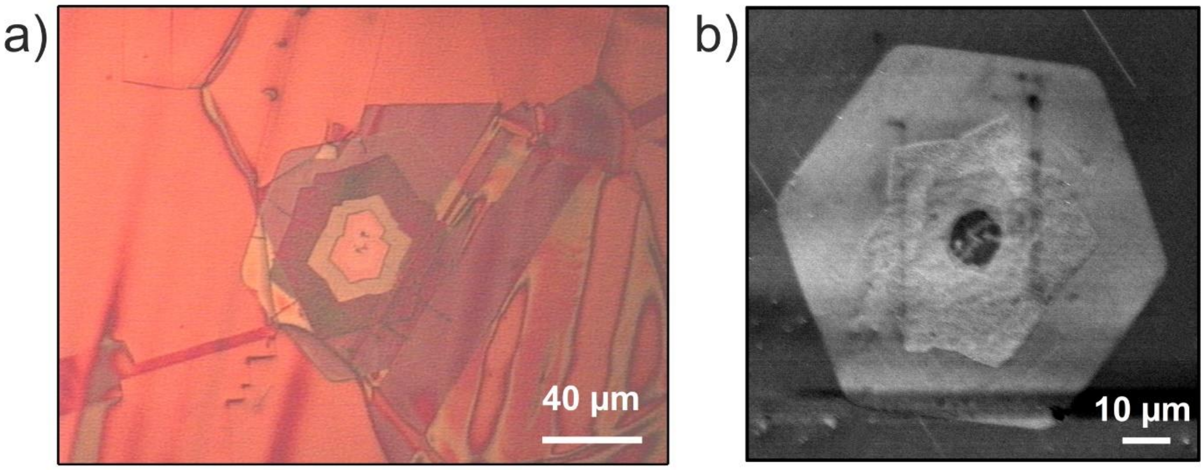

Figure S3. Graphene flakes imaged by optical and secondary electron microscopy after chemical vapor deposition (CVD) growth and the subsequent oxygen exposure of 5075 L at 950 °C in the quartz tube reactor. a) Optical microscopy (image size: 220 µm x 160 µm): The color change of the Cu foil under graphene clearly indicates the stepwise increasing layer thickness of the flake. b) Secondary electron microscopy of a graphene flake with stepwise increasing layer thickness acquired at 20 kV accelerating voltage (image size: 95 µm x 95 µm). Note that the optical image resolves a compact, hexagonally shaped island increasing in layer thickness in a wedding cake morphology with step edges aligned with respect to the ones of the lower lying graphene layer. The SEM data reveals a compact hexagonally shaped graphene layer, followed by a star-shaped layer. The star-shaped layer features step edges that are rotated by 30° compared to those of the lower layer on the left side of the flake. The dark vertical lines observed in the SEM image of the flake are a result of prolonged electron irradiation.

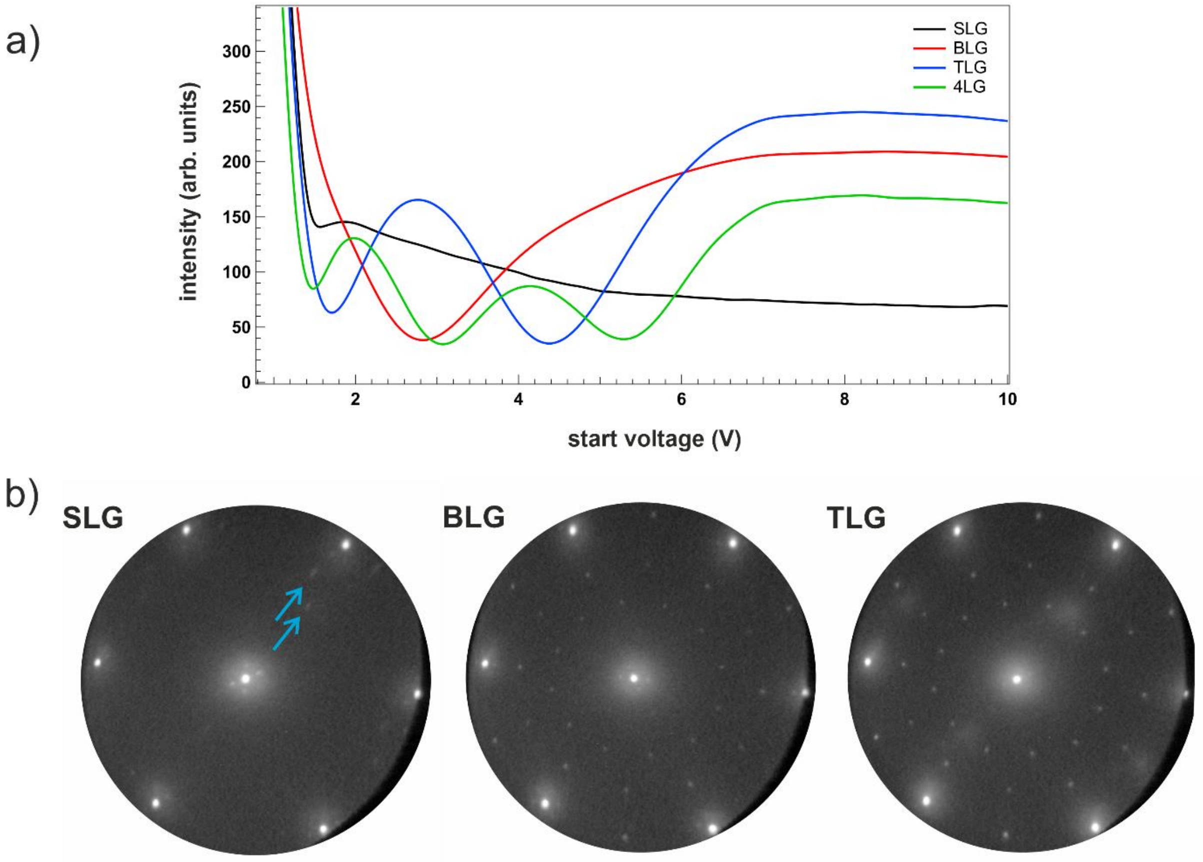

Figure S4. Additional data to Figure 3 of the main text. a) Electron reflectivity curves extracted from bright field LEEM images of SLG, BLG, TLG and 4 LG areas of the imaged graphene flake. The data clearly show that the flake remained intact and increased stepwise its thickness from a single graphene layer (SLG) to 4 layers. b) LEED patterns at 47 eV reveal the diffraction pattern of SLG, BLG and TLG. The weak spots in addition to the hexagonal pattern of the topmost layer indicate the stepwise stacking of 30° rotated graphene layers in BLG and TLG. The data evidence an intact graphene flake with inverted wedding cake morphology. Blue arrows point to additional diffraction spots on the SLG region which move in k-space when increasing the electron kinetic energy. Such spots are indicative for a Cu foil with the stepwise sequence of aligned and inclined facets on the Cu foil which account for the striped appearance of the real space LEEM images.

Figure S4 displays additional data to the images shown in Figure 3 of the main text characterizing the second sample after gentle degassing at 155°C overnight. The data displayed was acquired at room temperature. So-called LEEM-I(V) data were extracted from bright-field LEEM images of the graphene flake areas with SLG, BLG, TLG and 4LG thickness and are displayed in Figure S3a. The curves reflect the electron reflectivity of the (0,0) diffraction spot. The characteristic intensity oscillations at start voltages between 0 and 8 V with n dips in the reflectivity curve indicating intact graphene with a thickness of n+1 graphene layers.[3]

Figure S4b displays LEED patterns acquired at 47 eV from selected areas of the flake with SLG, BLG and TLG thickness. All patterns show the hexagonal diffraction pattern of an intact graphene lattice in the topmost layer. The weak additional spots in BLG and TLG originate from double scattering in the stacked 30° rotated layers of the graphene flake in regions with increasing thickness. Their low intensity evidence that these layers reside below the topmost layer indicating the known inverse wedding cake morphology of the graphene flake.[7]

Close inspection of the graphene flake shown in Figure 3 of the main text clearly indicates the alternating sequence of a compact hexagonal shape followed by a star-shaped island with increasing layer thickness like the island shape shown in Figure S1. The observed island step alignment with step edges rotated by 30° supports the stepwise stacking of 30° rotated layers.

So far, diffraction spots have been discussed that do not move in k-space when varying the start voltage (STV) which resembles the electron kinetic energy. Such spots originate from electron diffraction from a surface plane to which the microscope is aligned. Instead, interference of electrons reflected from an inclined surface plane leads to diffraction spots which move unidirectionally in k-space when increasing the start voltage. Such spots are observed in the diffraction pattern of SLG in Figure S4b where the propagation direction with increasing kinetic energy of the additional spots is indicated by blue arrows. This finding proves that the grown graphene flake resides on a support that consists of a sequence of an aligned surface plane followed by another, inclined one in a staircase morphology.[4, 6] This staircase morphology accounts for the stripe-appearance of the graphene flake in Figure 3 of the main text. The stripes (and thus the staircase morphology) under the imaged graphene flake are not only laterally resolved in LEEM images but also appear in real space XPEEM data. As discussed in the main text and also seen in Figures S5, the stripe contrast in photoelectron emission images and the observation of islands along these stripes can be traced back to silica formation underneath the graphene layer.

Figure S5 displays the C 1s, Si 2p, O 1s and Cu 3p XPEEM data acquired at the graphene flake edge, showing the bare Cu on the right lower part and the SLG part of the graphene flake on the left. The spectra displayed in the topmost row were extracted from regions of interest in the imaged area as indicated in the graph. Two lower rows of Figure S5 compile several images of the acquired XPEEM image stack from inset 1 of Figure 3 of the main text which reflect the photoelectron emission yield at the indicated energies (a, b, and c) in the corresponding spectra. As expected, the dosing of 5075 L O_2 at 950 °C in the CVD reactor dragged the bulk-dissolved Si to the surface of the uncovered Cu foil and induced silica formation as evidenced by the Si 2p and the O 1s core level at about 103 eV and 533 eV, respectively.[2] The silica formation did not proceed homogeneously but took place by island nucleation and growth. The islands are readily seen in the Si 2p-a and O 1s-a images which were taken at the kinetic energy (a) marked in the respective spectrum.

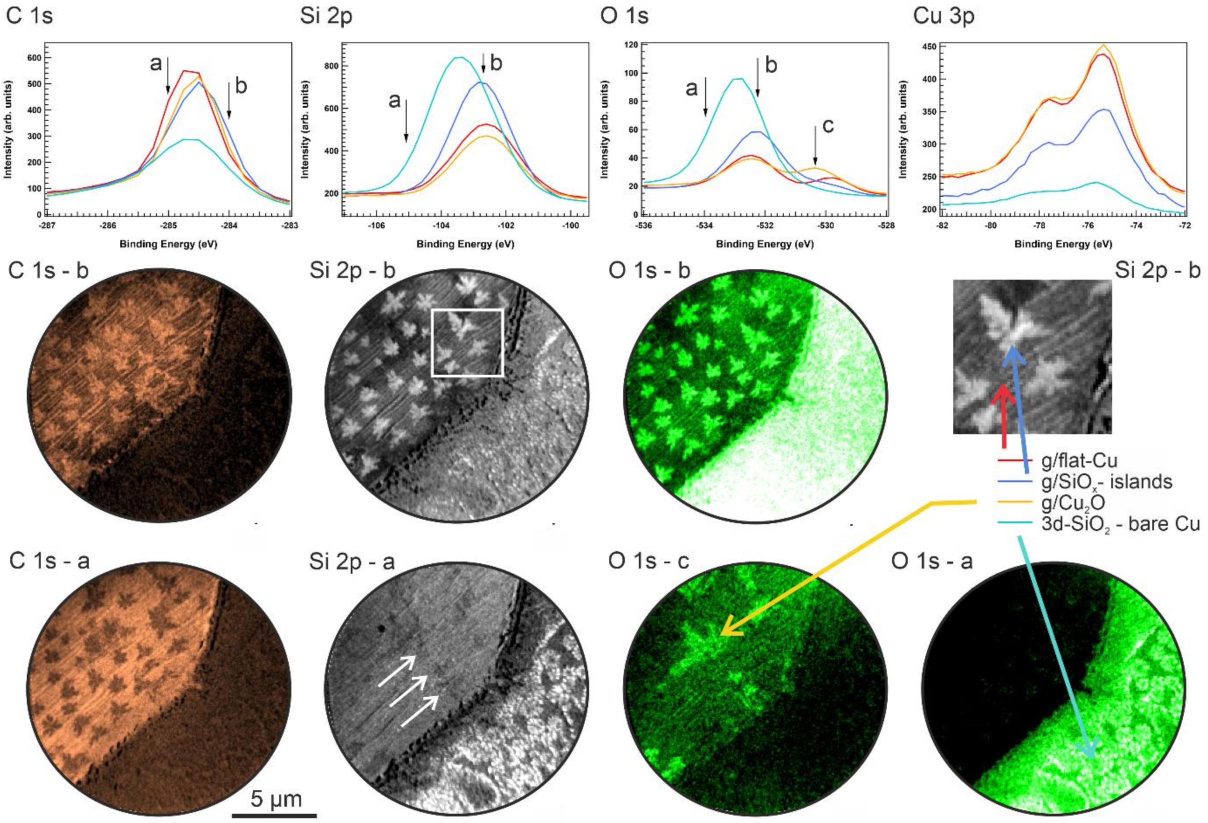

Figure S5. XPEEM data taken by collecting C 1s, Si 2p, Cu 3p and O 1s photoelectrons from graphene at the boundary to the uncovered Cu foil at position "1" in Figure 3. a) XP spectra extracted from 4 different regions of interest in the imaged area. b) XPEEM images reflect the spatially resolved photoelectron intensity at the indicated kinetic energies in the spectra. The oxygen treatment of the sample induced the formation of thick 3-dimensional silica islands on the Cu foil surrounding the graphene flake. However, the intercalation of a substantial amount of silica is also observed underneath graphene. Four regions of interest with different chemical properties can be identified with the help of the acquired spectra. Outside the graphene flake: 3-dimensional SiO_2 islands (3d-SiO_2 - bare Cu), underneath graphene: SiO_x islands with triangular fractal shape (g/SiO_x-islands), silica-covered Cu with striped appearance (g/flat-Cu) (see zoomed area of the Si 2p-b image) and patches along graphene wrinkles (see arrows in Si 2p-a) with enhanced amount of copper oxide (g/Cu_2O) (see O 1s-c image). Note that the contrast also appears in C 1s images because the intercalated species induce an energy shift of the C 1s photoelectrons towards lower binding energy. Photon energies: C 1s data: 400 eV, Cu 3p and Si 2p data: 260 eV, O 1s data: 640 eV.

The Si 2p and O 1s photoemission signal intensity acquired from islands outside the graphene flake exceeds the one taken from the 2-dimensional silica reference structure phase on Cu(111) by a factor of about 1.5 (see [10-12]). We thus identify the islands outside the graphene flake as 3-dimensional SiO_2 islands. This assignment is in accordance with the Si 2p and O 1s peak energies of 103.8 eV and 533 eV, respectively.[13, 14] Also, the observed strong signal damping of the Cu 3p photoemission outside the graphene flake supports the assignment of 3-dimensional SiO_2 islands. Note that the silica-covered Cu foil surrounding the graphene flake is not carbon-free because the silica-covered Cu foil adsorbed carbon contaminants during exposure to air after the treatment in the CVD reactor. This contaminant is not removed after transport to vacuum and degassing.

The oxygen-induced silica formation takes place not only outside of but also underneath the graphene flake. The contrast of the Si 2p - b image (taken at 102.8 eV) clearly resolves triangular islands with a

fractal shape on the region covered by SLG. This contrast is repeated in the O 1s - b image taken at 532.4 eV. Thus, we again assign the contrast to the formation of silica islands on the SLG-covered flake area (g/SiO_x-islands, a magnified Si 2p-b image is placed as inset in Figure S5). The slightly lower binding energy of the Si 2p and O 1s core level at 102.2 eV and 531.7 eV, respectively, indicates silica at an oxidation state lower than +4 as observed in thin silica films on Cu.

The Si 2p and the O 1s core level peak intensities can be quantified by comparison to the photoemission intensity of a 2-dimensional monolayer silica phase on Cu(111) as reference.[10-12] The Si 2p and O 1s intensities observed on the fractally shaped silica islands below SLG close to the edge of the graphene flake (see Figure S5) equal the ones obtained from the 2-dim silica reference phase. In contrast, the Si 2p and O 1s photoemission yield from the Cu foil area surrounding the fractal islands (g/flat-Cu) amounts to approximately 2/3 of the reference value. Keeping in mind that only 1/3 of the emitted photoelectrons at 100-160 eV kinetic energy pass the covering SLG layer,[15, 16] the amount of intercalated silica should equal to three times the one of monolayer silica in case of the fractal islands (SLG/SiO_x-islands) and to the average concentration of bilayer silica on the striped flat Cu surface surrounding the fractal islands below SLG (SLG/flat-Cu) taking into account the large inelastic mean free path of electrons in silica.[17]

The almost equal C 1s photoemission yield observed on both phases below SLG (i.e., g/SiO_x-islands and g/flat-Cu) indicates an intact covering graphene film which justifies the calculation of the signal-damping effect. Close inspection of the Si 2p - b and O 1s - b XPEEM images reveals a stripe contrast of the Cu foil area surrounding the fractal silica islands. The slight contrast variation of the stripes in the Si 2p and O 1s data indicates regions with different silica loading (see zoomed area of the Si 2p-b image). Knowing that the striped appearance of the Cu foil under graphene covered regions stems from a faceted Cu foil with staircase morphology of differently inclined surface facets, we can relate the varying photoemission intensity to a variation of the silica loading on the different facets (see discussion of Figure 5 of the main text of the manuscript and Figure S6 below).

Interestingly, the O 1s spectra show a peak at 532.4 eV relating to silica but also reveal the presence of an O 1s component at a binding energy close to 530 eV, which can be attributed to adsorbed oxygen or oxygen atoms in an oxidic environment. Tuning the photoelectron energy to 530.3 eV (indicated by c) in the O 1s spectrum) leads to the O 1s - c image which resolves areas with increased O 1s intensity. Since in these areas, a slightly lower Si 2p intensity and a weaker silica O 1s component at about 530 eV are observed, we attribute such areas to Cu_2O which formed at cost of silica. Note that the assignment of the O 1s species within the energy range between 529.6 eV and 530.4 eV to Cu_2O is compatible with literature data,[18] although also the attribution to an adsorbed species is possible as similar O 1s binding energies are observed for O_{ad}.[19] Close inspection of the Si 2p-a image reveals a faint dark line (see arrows) which is caused by a non-collapsed wrinkle almost invisible in the C 1s images. Comparison with the O 1s-c images shows that the identified Cu_2O areas developed along this wrinkle in the graphene flake. We relate the formation of Cu_2O patches to the contact to air before characterization in the SPELEEM instrument because copper oxide is thermodynamically unstable during oxygen dosing at 900 °C and thus cannot form during this treatment. Instead, during contact with air, gas phase oxygen rapidly reaches the Cu foil along wrinkles and trigger the formation of Cu-oxide while silicon segregation followed by silica formation does not take place at room temperature.

Although different in peak shape, the C 1s sum intensity is found to be almost constant in every part of the SLG flake region (g/flat-Cu, g/SiO_x-islands, and g/Cu_2O). This finding indicates that the covering SLG layer remained intact during oxygen exposure in the reactor treatment and during contact with air. However, the C 1s XPEEM images provide spatial contrast when tuning the photoelectron energy to the high (a) or low binding energy side (b) of the C 1s peak. Upon switching from C 1s - a to C 1s - b,

contrast reversal is observed, with the contrast correlating to the one observed in the Si 2p - b and O 1s - b images. I.e., while not changing in integral intensity, the C 1s peak shifts towards lower binding energy if oxygen is intercalated underneath the graphene layer where it is converted to silica. Oxygen-induced C 1s peak shifts were observed by non-spatially resolving XPS[20] or spatially resolved which could be used to image the onset of oxygen intercalation and conversion to silica.[2] The peak shift was related to the electronic n-doping of the graphene layer on Cu, which is diminished where oxygen intercalates underneath graphene; this was also observed in ARPES data.[21] As seen already in Figure 1 and 2 of the main text, n-doping of SLG is confirmed on metallic Cu(111) and the C 1s shift towards lower binding energy would occur wherever intercalated silica decouples the graphene layer from the Cu foil support and diminishes the n-doping level.

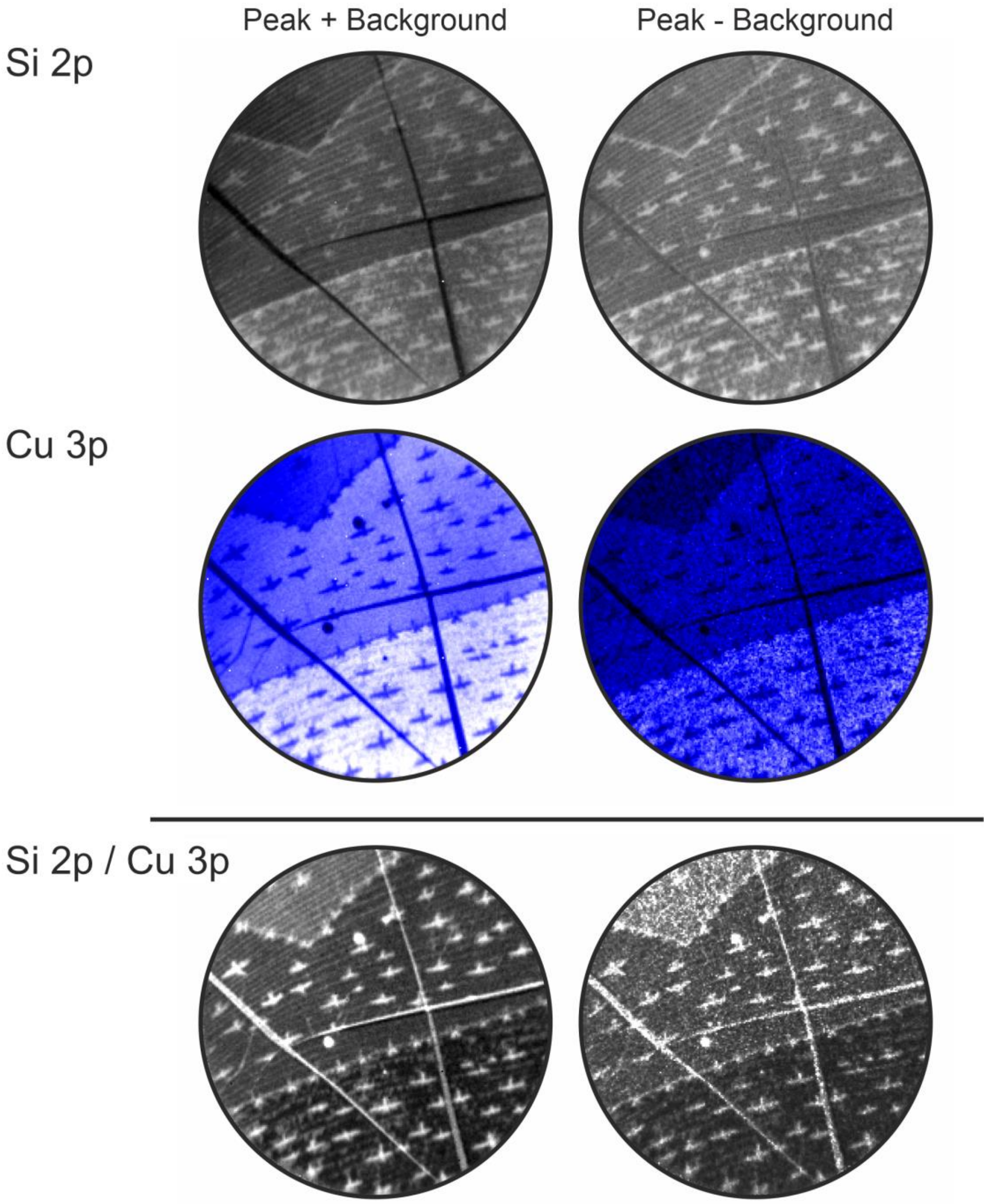

Figure S6. XPEEM data before and after background correction and the effect on the quality of the resulting Si 2p /Cu 3p ratio image (see Figure 5 of the main text).

During the discussion of Figure 5 of the main text it was stated that a Si 2p / Cu 3p ratio image removes the geometric contrast from XPEEM images.[22] Since also the effect of a covering graphene layer is removed the data were used to prove that intercalated silica islands decorate the faceted Cu foil and that the island distribution is affected by the thickness of the covering graphene film. Figure 5 of the main text displays the ratio image where the Si 2p-nominator and the Cu 3p- denominator contained photoelectron background intensity. Of course, the background intensity can be removed before calculating the ratio image. However, considerable amount of noise is introduced in the image that obscures small lateral features in the image. The background corrected ratio image delivers grey scale numbers that fit the photoemission intensities shown in the charts of Figure 4. Nevertheless, the qualitative contrast level of both images does not change. This is why only the noise-free image is shown in the main text. Figure S6 shows the entire data set. Note that the XPEEM images compiled in Figure 4 of the main text are background-corrected so that the Si 2p and Cu 3p images of the right column in Figure S6 are identical to the ones of Figure 4.

During the discussion of the data presented in Figure 4 and 5, it was mentioned that other silica geometries may be thought that deliver a regular C 1s and Cu 3p intensity scaling but lead to the irregular Si 2p and O 1s signal decrease deviating from the expected signal damping due to the covering SLG, BLG, TLG and 4LG graphene layer:

a) Of course, silica covered regions of the foil might completely uncovered or not covered by more than a single graphene layer even in the BLG, TLG and 4LG regions of the flake so that the Si 2p and O 1s photoemission signal would remain unaffected. However, the regular scaling of the C 1s and the Cu 3p intensity excludes such a picture to a large extent. Also, the contrast observed in Figure 5 and S6 is incompatible with such a picture as it is independent of the thickness of covering layers. However, the large, cross-shaped islands (g/SiO_x-islands) might be only partially covered by graphene since in such areas, minor deviations of the photoemission intensity scaling are locally observed.

b) A second scenario explaining a too high Si 2p and O 1s photoemission yield is the presence of silica intercalated between stacked graphene layers in the BLG, TLG and 4LG thick regions of the graphene flake so that the respective emission intensities are less damped than expected. Such a configuration would lead to the observed contrast in Figure 5 and S6. However, this possibility seems to be unlikely because there is no thermodynamic driving force for silica to diffuse from the silica-Cu interface in between graphene layers as this cost energy. In addition, the presented LEED and ARPES data in the main manuscript show the patterns of coherently stacked graphene layers which would be lost when vertically separating subsequent graphene layer on the nm length scale.

c) Finally, assuming silica resident on top of graphene flake will lead to a Si 2p and O 1s signal independent of the thickness of the graphene underneath. However, the formation of the silica by oxygen induced segregation from dissolved silicon must start at the Cu interface below graphene making this geometry highly unlikely. However, one could imagine that during the oxygen exposure at 900 °C during second treatment in the CVD reactor, a minor amount of SiO species were removed from the quartz reactor wall that landed on top of the synthesized graphene flake, despite the fact that SiO removal requires a reductive hydrogen atmosphere. Assuming all such unlikely processes, the silica loading ontop of the graphene flake should be uniform because of the inverted wedding cake geometry of the flake. (The outermost layer of the flake is the carbon network of the SLG-layer.) Since in this case, Figure 5 would deliver no contrast in disagreement with the experimental finding, we can safely disregard also this unlikely process.

Suitable reactor treatment parameters enabling silica intercalation and graphene decoupling

The suitable degree of oxygen exposure after CVD growth of graphene on Si-loaded Cu foils was investigated with the help of Raman spectroscopy and optical microscopy. The left panels of Figure S7a), b) and c) compile optical microscopy data of graphene-covered areas (left side of each image) in the vicinity of the graphene-free Cu foil surface (right image part).

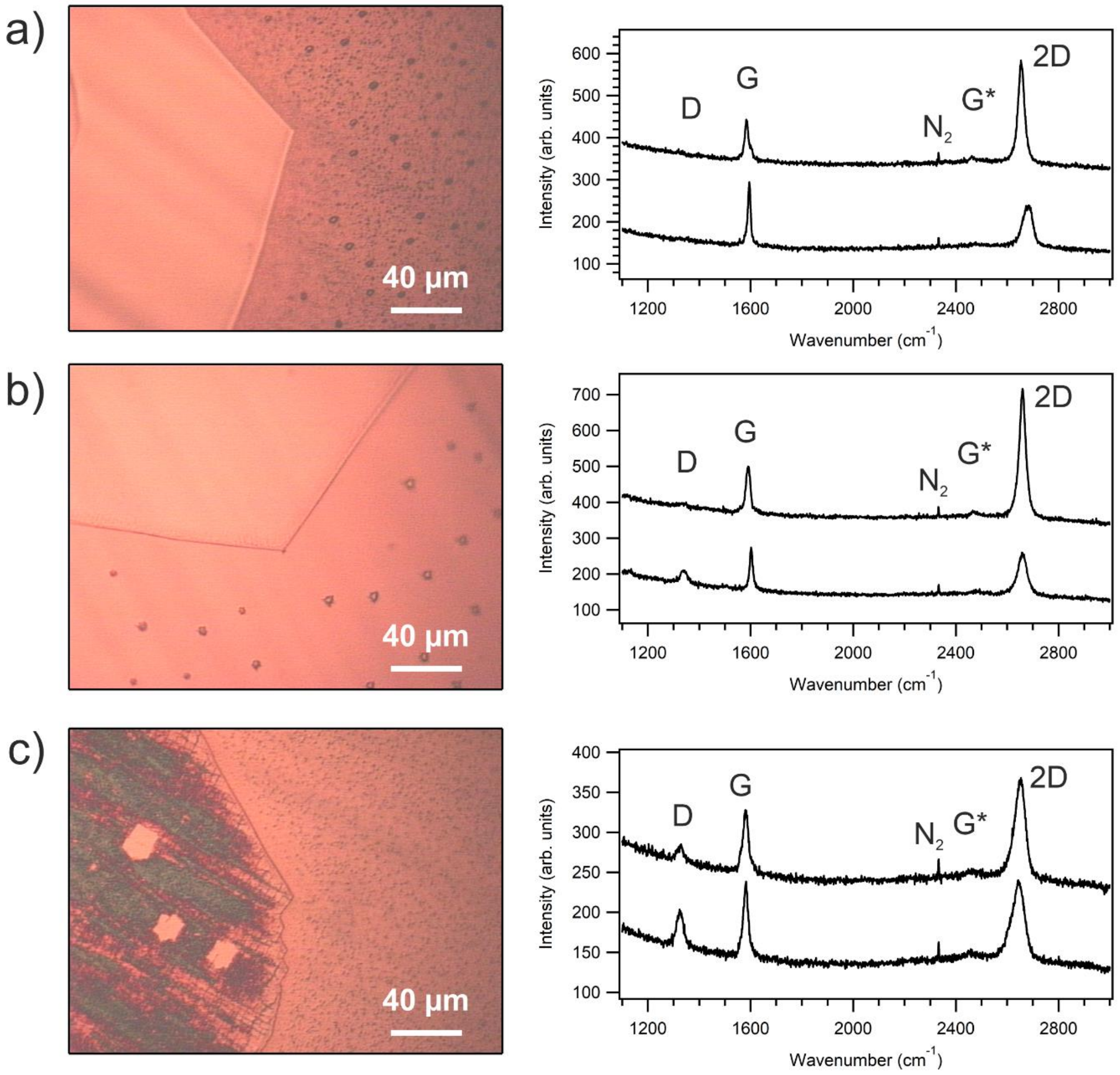

Figure S7. Optical microscopy (left panels, image size 220 µm x 160 µm) and Raman spectroscopy data (right panels) of CVD-grown graphene on Si-loaded Cu foils after subsequent O_2 exposure to 10500 L at 950 °C (a), 6800 L at 1075 °C (b) and 20500 L at 850 °C (c). The Raman spectra were acquired using a Horiba Jobin Yvon HR800 UV spectrometer coupled to a HeNe-Laser (wavelength 632.8 nm), resolving the D-, G-, G*-, and 2D-band of graphene[23] and the vibration mode of molecular nitrogen in air.

As shown in Figure S5, the oxygen impingement on the bare Cu surface induces the segregation of dissolved silicon and its conversion to thick silica islands. These islands are resolved in the compiled optical microscopy data of Figure S7 and show an island size increase when raising the

temperature during oxygen exposure from T = 850°C (c) to T = 950 °C (a) and T = 1075 °C (b). This finding indicates the expected increase in surface mobility at elevated temperature.

The observed silica formation underneath graphene (see Ref.[2], Figures S5, S6, and Figures 4 and 5 of the main manuscript text) is not resolved by optical microscopy. In Figures S7a) and b), the graphene-covered region appears featureless. However, in Figure S7c), the graphene-covered region is imaged with a dark red contrast, which hosts 3 small featureless islands. We assign the dark red straight lines in the large flake to wrinkles in the graphene layer, which were shown to be channels where molecular oxygen from the gas phase can reach the Cu surface and trigger the segregation mediated formation of intercalated silica.[2]

The color change of the Cu foil from orange to dark red is assigned to the degree of oxygen intercalation and Cu oxide formation, which is a competing process of silica intercalation (see Figure S5). The three small featureless islands are assigned to areas with increased graphene thickness where oxygen intercalation and Cu oxide formation take place at a reduced rate (see main text of the manuscript). The contrast assignment of the optical microscopy data agrees with the acquired Raman data shown in the right panels of Figure S7a), b) and c) and can be related to the conditions applied to induce silica intercalation.

In Figure S7a), the CVD-grown graphene flakes were exposed to 10500 L O_2 at 950 °C and Raman spectra with the signature of SLG (top spectrum) and BLG (bottom spectrum) can be recorded. Note that the displayed spectra show no D-band, proving defect-free graphene.[23] It should be noted that flakes with a vanishingly small D-band were also found on the sample, especially when recording the spectra close to the flake edge. The same is observed when acquiring Raman data from CVD-grown graphene on Cu after exposure to 6800 L O_2 at 1075 °C. Again, defect-induced D-bands below or slightly above the detection limit are recorded as shown in the right panel of Figure S7b). However, when raising the O_2 exposure to 20500 L at 850 °C (see right panel of Figure S7c), Raman spectra without the appearance of a D-band are not recorded anymore. Two processes may contribute to the generation of defects in the covering graphene film when inducing silica intercalation. Obviously, the enlarged oxygen dosing of 20500 L enhances the probability that the impinging oxygen attacks the graphene layer. However, lowering the temperature may also contribute to defect formation as the oxygen exposure at 850 °C will slow down the Si segregation and silica formation. This process is known to act as an oxygen scavenger[2] resulting in an increased amount of reactive oxygen on the Cu foil during oxygen exposure, promoting Cu oxidation and the local removal of the covering graphene layer.

It is difficult to judge which effect is more important because the defect formation kinetics are also affected by the surface termination of the local crystalline grain and the degree of Si loading of the Cu support foil. However, the exposure of CVD-grown graphene to 5075 L O_2 at 950 °C applied for the treatment of sample 2 amounts to about half the dose used for the preparation of the sample shown in Figure S7a). As this should be a safe choice for the formation of almost defect-free graphene on Cu, sample 2 is expected to deliver data of highly crystalline graphene, electronically decoupled from the Cu support by intercalating silica, as shown in the main text of the manuscript.

REFERENCES

(1) Reckinger, N.; Tang, X.; Joucken, F.; Lajaunie, L.; Arenal, R.; Dubois, E.; Hackens, B.; Henrard, L.; Colomer, J.-F. Oxidation-assisted graphene heteroepitaxy on copper foil. *Nanoscale* **2016**, *8* (44), 18751-18759. DOI: 10.1039/C6NR02936A.

(2) Kratky, T.; Leidinger, P.; Zeller, P.; Kraus, J.; Genuzio, F.; Jugovac, M.; Sala, A.; Menteş, T. O.; Locatelli, A.; Günther, S. The fate of graphene on copper: Intercalation / de-intercalation processes and the role of silicon. *Carbon* **2024**, *226*, 119172. DOI: 10.1016/j.carbon.2024.119172.

(3) Srivastava, N.; Gao, Q.; Widom, M.; Feenstra, R. M.; Nie, S.; McCarty, K. F.; Vlassiouk, I. V. Low-energy electron reflectivity of graphene on copper and other substrates. *Physical Review B* **2013**, *87* (24), 245414.

(4) Leidinger, P.; Kraus, J.; Kratky, T.; Zeller, P.; Menteş, T. O.; Genuzio, F.; Locatelli, A.; Günther, S. Toward the perfect membrane material for environmental x-ray photoelectron spectroscopy. *Journal of Physics D: Applied Physics* **2021**, *54* (23), 234001. DOI: 10.1088/1361-6463/abe743.

(5) Zeller, P.; Ma, X.; Günther, S. Indexing moiré patterns of metal-supported graphene and related systems: strategies and pitfalls. *New Journal of Physics* **2017**, *19* (1), 013015. DOI: 10.1088/1367-2630/aa53c8.

(6) Kraus, J.; Boecklein, S.; Reichelt, R.; Guenther, S.; Santos, B.; Mentes, T. O.; Locatelli, A. Towards the perfect graphene membrane? - Improvement and limits during formation of high quality graphene grown on Cu-foils. *Carbon* **2013**, *64*, 377-390. DOI: 10.1016/j.carbon.2013.07.090.

(7) Nie, S.; Wu, W.; Xing, S.; Yu, Q.; Bao, J.; Pei, S.-s.; McCarty, K. F. Growth from below: bilayer graphene on copper by chemical vapor deposition. *New Journal of Physics* **2012**, *14* (9), 093028. DOI: 10.1088/1367-2630/14/9/093028.

(8) Bauer, E. Multiple scattering versus superstructures in low energy electron diffraction. *Surface Science* **1967**, *7* (3), 351-364. DOI: 10.1016/0039-6028(67)90026-X.

(9) Lisi, N.; Dikonimos, T.; Buonocore, F.; Pittori, M.; Mazzaro, R.; Rizzoli, R.; Marras, S.; Capasso, A. Contamination-free graphene by chemical vapor deposition in quartz furnaces. *Scientific Reports* **2017**, *7* (1), 9927. DOI: 10.1038/s41598-017-09811-z.

(10) Navarro, J. J.; Tosoni, S.; Bruce, J. P.; Chaves, L.; Heyde, M.; Pacchioni, G.; Roldan Cuenya, B. Structure of a Silica Thin Film on Oxidized Cu(111): Conservation of the Honeycomb Lattice and Role of the Interlayer. *The Journal of Physical Chemistry C* **2020**, *124* (38), 20942-20949. DOI: 10.1021/acs.jpcc.0c05463.

(11) Xu, J.; Mu, C.; Chen, M. Structure and Properties of Ultrathin SiOx Films on Cu(111). *Langmuir* **2022**, *38* (37), 11414-11420. DOI: 10.1021/acs.langmuir.2c01701.

(12) Kratky, T.; Leidinger, P.; Genuzio, F.; Menteş, T. O.; Locatelli, A.; Günther, S. Commensurability of Monolayer Silica on Cu(111). *The Journal of Physical Chemistry C* **2024**, *128* (17), 7235-7241. DOI: 10.1021/acs.jpcc.3c08360.

(13) Alfonsetti, R.; Lozzi, L.; Passacantando, M.; Picozzi, P.; Santucci, S. XPS studies on SiOx thin films. *Applied Surface Science* **1993**, *70-71*, 222-225. DOI: 10.1016/0169-4332(93)90431-a.

(14) Kerkhof, F. P. J. M.; Moulijn, J. A.; Heeres, A. The XPS spectra of the metathesis catalyst tungsten oxide on silica gel. *Journal of Electron Spectroscopy and Related Phenomena* **1978**, *14* (6), 453-466. DOI: 10.1016/0368-2048(78)87004-2.

(15) Apponi, A.; Convertino, D.; Mishra, N.; Coletti, C.; Iodice, M.; Frasconi, F.; Pilo, F.; Blaj, N. S.; Paoloni, D.; Rago, I.; et al. Transmission through graphene of electrons in the 30 – 900 eV range. *Carbon* **2024**, *216*, 118502. DOI: 10.1016/j.carbon.2023.118502.

(16) Kraus, J.; Reichelt, R.; Gunther, S.; Gregoratti, L.; Amati, M.; Kiskinova, M.; Yulaev, A.; Vlassiouk, I.; Kolmakov, A. Photoelectron spectroscopy of wet and gaseous samples through graphene membranes. *Nanoscale* **2014**, *6* (23), 14394-14403, 10.1039/C4NR03561E. DOI: 10.1039/C4NR03561E.

(17) Astašauskas, V.; Bellissimo, A.; Kuksa, P.; Tomastik, C.; Kalbe, H.; Werner, W. S. M. Optical and electronic properties of amorphous silicon dioxide by single and double electron spectroscopy. *Journal of Electron Spectroscopy and Related Phenomena* **2020**, *241*, 146829. DOI: https://doi.org/10.1016/j.elspec.2019.02.008.
(18) Deroubaix, G.; Marcus, P. X-ray photoelectron spectroscopy analysis of copper and zinc oxides and sulphides. *Surface and Interface Analysis* **1992**, *18* (1), 39-46. DOI: 10.1002/sia.740180107.
(19) Baddorf, A. P.; Wendelken, J. F. High coverages of oxygen on Cu(110) investigated with XPS, LEED, and HREELS. *Surface Science* **1991**, *256* (3), 264-271. DOI: 10.1016/0039-6028(91)90869-T.
(20) Blume, R.; Kidambi, P. R.; Bayer, B. C.; Weatherup, R. S.; Wang, Z. J.; Weinberg, G.; Willinger, M. G.; Greiner, M.; Hofmann, S.; Knop-Gericke, A.; et al. The influence of intercalated oxygen on the properties of graphene on polycrystalline Cu under various environmental conditions. *Phys Chem Chem Phys* **2014**, *16* (47), 25989-26003. DOI: 10.1039/c4cp04025b.
(21) Gottardi, S.; Müller, K.; Bignardi, L.; Moreno-López, J. C.; Pham, T. A.; Ivashenko, O.; Yablonskikh, M.; Barinov, A.; Björk, J.; Rudolf, P.; et al. Comparing Graphene Growth on Cu(111) versus Oxidized Cu(111). *Nano Letters* **2015**, *15* (2), 917-922. DOI: 10.1021/nl5036463.
(22) Günther, S.; Kolmakov, A.; Kovac, J.; Kiskinova, M. Artefact formation in scanning photoelectron emission microscopy. *Ultramicroscopy* **1998**, *75* (1), 35-51. DOI: 10.1016/s0304-3991(98)00047-3.
(23) Malard, L. M.; Pimenta, M. A.; Dresselhaus, G.; Dresselhaus, M. S. Raman spectroscopy in graphene. *Physics Reports* **2009**, *473* (5–6), 51-87. DOI: http://dx.doi.org/10.1016/j.physrep.2009.02.003.
